# In utero gene expression in the *Slc39a8(neo/neo*) knockdown mouse

**DOI:** 10.1038/s41598-018-29109-y

**Published:** 2018-07-16

**Authors:** Jing Chen, Marina Gálvez-Peralta, Xiang Zhang, Jingyuan Deng, Zijuan Liu, Daniel W. Nebert

**Affiliations:** 1 Division of Biomedical Informatics, Cincinnati Children’s Hospital Medical Center, Cincinnati, Ohio, 45229 USA; 2 Department of Environmental Health, University of Cincinnati College of Medicine, Cincinnati, Ohio, 45267 USA; 3 Department of Biological Sciences, Oakland University, Rochester, MI 48309 USA; 4 Present Address: Department of Pharmaceutical Sciences, West Virginia University Medical Center, Morgantown, WV 26506 USA; 5 Present Address: Amazon.com, Inc., Seattle, WA 98101 USA

## Abstract

*Slc39a*8 encodes ZIP8, a divalent cation/bicarbonate symporter expressed in pluripotent mouse embryonic stem cells, and therefore ubiquitous in adult tissues; ZIP8 influxes Zn^2+^, Mn^2+^ and Fe^2+^. *Slc39a8(neo/neo)* knockdown mice exhibit 10–15% of wild-type ZIP8 mRNA and protein levels, and show pleiotropic phenotype of stunted growth, neonatal lethality, multi-organ dysmorphogenesis, and dysregulated hematopoiesis manifested as severe anemia. Herein we performed RNA-seq analysis of gestational day (GD)13.5 yolk sac and placenta, and GD16.5 liver, kidney, lung, heart and cerebellum, comparing *Slc39a8(neo/neo)* with *Slc39a8*(+/+) wild-type. Meta-data analysis of differentially-expressed genes revealed 29 unique genes from all tissues — having enriched GO categories associated with hematopoiesis and hypoxia and KEGG categories of complement, response to infection, and coagulation cascade — consistent with dysregulated hematopoietic stem cell fate. Based on transcription factor (TF) profiles in the JASPAR database, and searching for TF-binding sites enriched by Pscan, we identified numerous genes encoding zinc-finger and other TFs associated with hematopoietic stem cell functions. We conclude that, in this mouse model, deficient ZIP8-mediated divalent cation transport affects zinc-finger (*e.g*. GATA proteins) and other TFs interacting with GATA proteins (*e.g*. TAL1), predominantly in yolk sac. These data strongly support the phenotype of dysmorphogenesis and anemia seen in *Slc39a8(neo/neo)* mice in utero.

## Introduction

Starting with a Cd^2+^-induced testicular necrosis phenotype and genetically “sensitive” vs “resistant” inbred mouse strains, this laboratory identified *Slc39a8* (encoding the ZIP8 transporter) as the major gene responsible for this trait^1–3^. ZIP8 functions as a Zn^2+^**/**(HCO_3_^−^)_2_, Mn^2+^**/**(HCO_3_^−^)_2_ or Cd^2+^**/**(HCO_3_^−^)_2_ symporter, in each case moving all three ions of the complex into the cell in an electroneutral manner^4^. ZIP8 has also been shown to import divalent iron and cobalt^5^. Moreover, ZIP8 transports selenium in the form of selenite into the cell — presumably as a Zn^2+^**/**(HCO_3_^−^)(HSeO_3_^−^) electroneutral complex^6^. In mammalian cell and *Xenopus* oocyte cultures, Mn^2+^ and Fe^2+^ are able to substitute for the Zn^2+^ cation^5,7,8^. It is possible that metal-ion specificity of ZIP8-mediated uptake in the intact animal might be dependent on organ and/or cell-type.

Developmentally, ZIP8 is expressed in mouse visceral endoderm at gestational day (GD)7.5 [ref.^9^], in the gastrula stage^10^, and in pluripotent embryonic stem cells^11^. These findings are compatible with the observation of early embryolethality, seen in *Slc39a8(−*/*−)* knockout mice when the gene is globally 100% ablated^12^.

During creation of a *Slc39a8* conditional knockout construct^12^, the *Frt*-flanked neomycin-resistance mini-gene *neo* (transcribed in reverse orientation) was inserted into intron 3, combined with *loxP* sites in introns 3 and 6. Fortuitous retention of the *neo* mini-cassette produced a unique hypomorph displaying 10–15% of wild-type levels of ZIP8 mRNA and protein in all tissues examined^1–3^. The *Slc39a8(neo/neo)* mouse has been extensively characterized: pleiotropic effects include stunted growth, neonatal lethality, shortened limbs and deformed skull, severe anemia, dysregulation of hematopoiesis, and multi-organ dysmorphogenesis; consistent with the severe anemia — striking decreases in size of placenta, and size and number of hematopoietic islands are seen in *Slc39a8(neo/neo)* GD13.5 yolk sac and placenta, as well as in GD16.5 liver^13^.

A *Slc39a8* inducible-global knockout and a *Slc39a8* hepatocyte-specific conditional knockout, created independently, exhibited striking decreases in Mn^2+^ concentrations in multiple organs tested; authors also found lowered levels of two Mn^2+^-dependent enzymes – arginase and β-1,4-galactosyltransferase activities – and evidence that hepatic ZIP8 rescues Mn^2+^ from bile and regulates whole-body Mn^2+^ homeostasis^14^. The *Slc39a8* global knockout shows striking cardiac extracellular matrix accumulation, suggesting that *Slc39a8* expression is essential for development of ventricular compaction^15^.

These mouse data^1–16^, summarized in Table 1, underscore the likely fundamental importance of ZIP8-mediated physiological functions in a large variety of tissues and cell-types. The gene was originally discovered in human monocytes^17^. More recently, a growing number of clinical studies of *SLC39A8* variants have shown the pleiotropic effect of deficient ZIP8 function: during early development^18–20^ and in liver^14,18,19^, kidney^21^, lung^22–25^, heart/cardiovascular system^21,26–31^, whole blood^32^, immune system^17,22–25^, brain^20,33,34^ including cerebellum^18,19^, eye^35^, gastrointestinal tract^36^, and musculoskeletal system^18,19,37,38^ (Table 1). In the present study, we chose to carry out RNA-seq analysis on seven tissues: GD13.5 yolk sac and placenta, plus GD16.5 fetal liver, kidney, lung, heart and cerebellum.

**Table 1:** Properties and phenotypes reported, to date, for mammalian *SLC39A8* gene.

Organ or system	Properties/Phenotypes of mammalian *SLC39A8* gene (and its encoded ZIP8 transporter)	References
Systemic	Mouse ZIP8 transports Zn^2+^, Mn^2+^, Cd^2+^/and probably Fe^2+^ and Co^2+^ — each presumanly as a M^++^/(HCO_3_^−^)_2_ electroneutral complex, moving ions into the cell; *Slc39a8(neo/neo)* knockdown fetus exhibits stunted growth, and neonatal lethality; Human SLC39A8 regulates manganese homeostasis and manganese-dependent enzyme activities, posttranslational glycosylation, and causes mitochondrial disorders; Mouse ZIP8 is required for Mn^2+^-dependent enzyme activities; Human *SLC39A8* variant associated with increased body mass index	^2, 4, 5, 7, 13, 14, 19, 20, 32, 79^
Developmental	Mouse *Slc39a8* expressed in visceral endoderm at gestational day (GD)7.5, in the gastrula at earlier development, and expressed in pluripotent embryonic stem cells; Mouse *Slc39a8(neo/neo)* global knockout is early embryo-lethal; *Slc39a8(neo/neo)* knockdown fetus exhibits multi-organ dysmorphogenesis and decreases in size of placenta	^9– 13, 18– 20, 80^
Liver	Mouse *Slc39a8(neo)* allele is associated with highly significantly decreased liver size; Mouse ZIP8 transports selenium, presumably as a Zn^2+^/(HCO_3_^−^)(HSeO_3_^−^) electroneutral complex; Mouse hepatic ZIP8 rescues Mn^2+^ from bile, regulates whole-body Mn^2+^ homeostasis, and modulates activity of Mn^2+^-dependent enzymes	^6, 13, 14, 18, 19^
Kidney	Mouse *Slc39a8(neo)* allele is associated with highly significantly decreased kidney size; Human *SLC39A8* variant associated with blood pressure	^13, 21^
Lung	Mouse *Slc39a8(neo)* allele is associated with highly significantly decreased lung size; Human ZIP8 participates in Zn^2+^-mediated cytoprotection in lung inflammation	^13, 22, 23^
Cardiovascular system	Mouse *Slc39a8(neo/neo)* knockdown fetus shows trend^a^ toward enlarged heart size; Human *SLC39A8* variant associated with increased serum lipid levels, regulation of blood pressure, and risk of coronary artery disease; Human *SLC39A8* variant associated with increased risk of acute coronary syndrome; Human *SLC39A8* variant associated with increased risk of increased atherosclerosis in smokers; Mouse *Slc39a8* is essential for cardiac ventricular compaction	^13, 15, 21, 26– 31^
Blood chemistry	Mouse *Slc39a8(neo/neo)* neonate shows significantly decreased total iron, total iron-binding capacity, serum ALT & AST levels,^b^ and serum triglyceride levels; Human *SLC39A8* variant associated with blood levels of toxic metals	^13, 32^
Hematological system	Mouse *Slc39a8(neo/neo)* knockdown fetus exhibits severe anemia and dysregulation of hematopoiesis; *Slc39a8(neo/neo)* GD13.5 yolk sac and placenta shows smaller size and number of hematopoietic islands; *Slc39a8(neo/neo)* GD16.5 liver reveals decreased size and number of hematopoietic islands	^13^
Immune system	ZIP8 originally discovered in human monocytes; Human ZIP8 participates in Zn^2+^-mediated immune response to inflammation, participates in innate immune response to endotoxin-induced macrophage inflammation, and host response in macrophages to *Mycbacterium tuberculosis*	^17, 22– 25^
Central nervous system	Mouse *Slc39a8(neo/neo)* knockdown fetus shows trend^a^ in smaller size of cerebrum and cerebellum; Human *SLC39A8* variant associated with increased risk of schizophrenia; Human *SLC39A8* variant associated with impaired intellectual ability and cerebellar atrophy	^13, 18– 20, 33, 34^
Eye	Human *SLC39A8* variant associated with retinal iron accumulation	^35^
Spleen	Mouse *Slc39a8(neo)* allele is associated with highly significantly decreased spleen size	^13^
Gastrointestinal tract	Human *SLC39A8* variant associated with Crohn disease and human gut microbiome composition	^36^
Musculoskeletal system	Mouse *Slc39a8(neo/neo)* knockdown fetus exhibits shortened limbs, and deformed skull; Human *SLC39A8* variant associated with pathogenesis of osteoarthritis; Human *SLC39A8* variant associated with severe limb malformations	^13, 18, 19, 37, 38^
Reproductive system	Mouse *Slc39a8* variant is associated with Cd^2+^-induced testicular necrosis	^1– 3, 16^

^a^The term “trend’” denotes *P*-value > 0.05 < 0.10.

^b^ALT, alanine aminotransferase (common measurement used to assess damage largely in liver); AST, aspartate aminotransferase (common measurement used to assess damage in heart, skeletal muscle, kidney and brain, as well as liver.

## Results

### Phenotype

**Figure 1:**
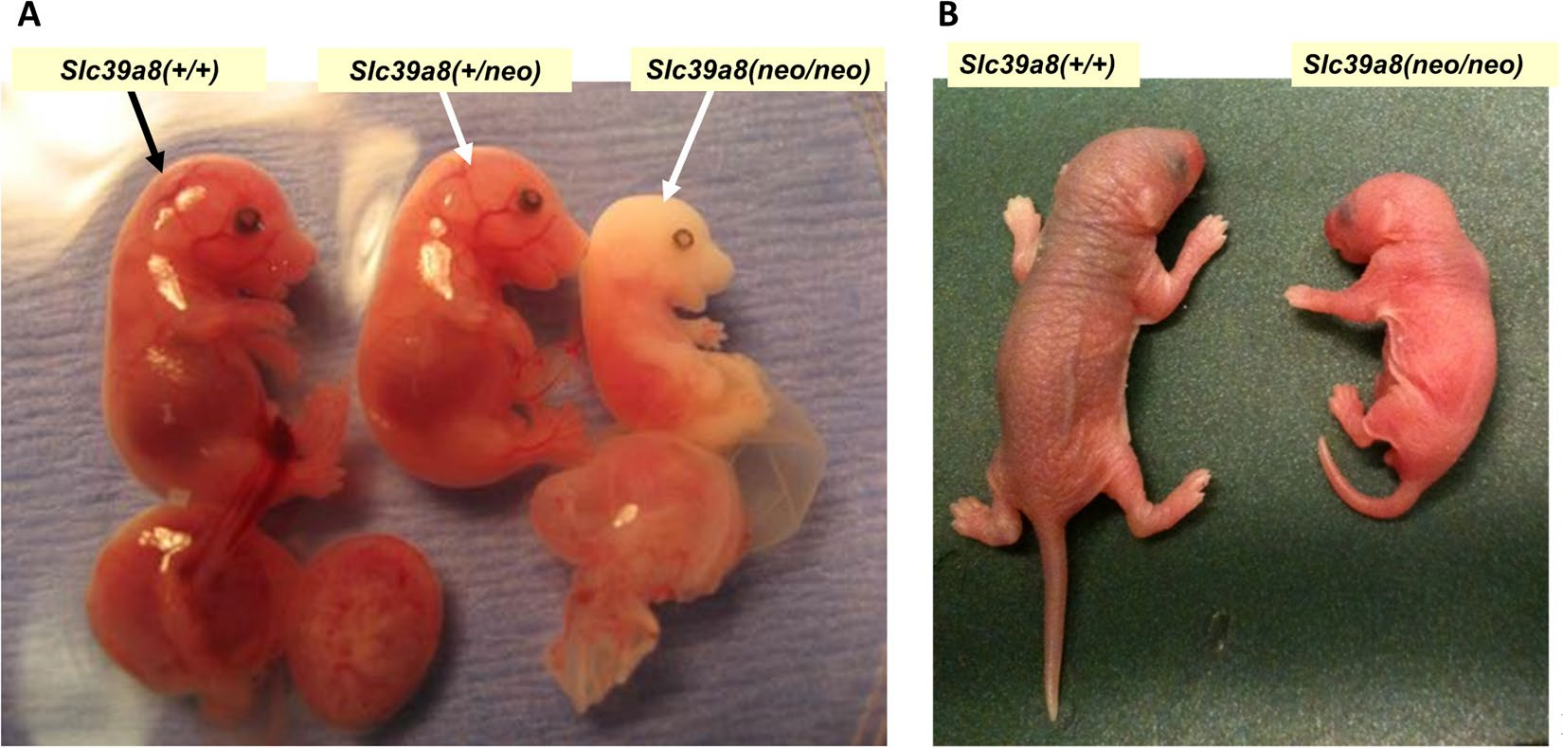
Phenotype of *Slc39a8*(+/+), *Slc39a8*(+*/neo)*, and *Slc39a8(neo/neo)* littermates. (**A**) GD16.5 pups with corresponding placentas below. *Slc39a8(neo/neo)* mice were remarkably abnormal – showing deformed skull and shortened limbs, as well as severe anemia, from the time during development that an embryonal sac can first be observed. (**B**) Newborns, postnatal day 1 – shortly before death of the *Slc39a8(neo/neo)* homozygote. Compared with *Slc39a8*(+/+) wild-type and *Slc39a8*(+*/neo)* heterozygotes that are pink in color and normal in size, the *Slc39a8(neo/neo)* littermates are extremely pale, show stunted growth, and deformed skulls and limbs. *Slc39a8(neo/neo)* liver, kidney, lung, spleen, cerebrum and cerebellum were all statistically significantly smaller in size than that in *Slc39a8*(+/+) or *Slc39a8*(+*/neo)* littermates^13^. For the RNA-seq analysis described herein, *Slc39a8*(+*/neo)* littermates were not studied.

Compared with *Slc39a8*(+/+) wild-type, the *Slc39a8(neo/neo)* fetuses and newborns were remarkably abnormal with dysmorphogenesis and severe anemia (Fig. 1); even placenta and yolk sac showed gross anemia^13^.

### Analysis of differentially-expressed genes in individual tissues

Differential-expression analysis started with low-expression filtering, following which 6,249 genes were removed and 17,163 genes retained (having CPM >1 in at least three samples). The number of significant differentially-expressed genes (Table 2) included 15 in yolk sac, two in placenta, one in liver, 13 in lung, but none in kidney, heart and cerebellum. Two genes (*Adh*7 and *Zbtb8b*) encode Zn^2+^-containing proteins. A heat map (Supplementary Data Figure S1) shows intensities of each gene expression.

**Table 2:** Genes differentially regulated with FDR < 0.1, when comparing *Slc39a8(neo/neo)* with *Slc39a8*(+/+) wild-type.

Gene symbol	Official gene name	Average expression (RPKM)^a^	Fold-Change	log_2_ Fold-Change	*P*_val_	*P*_adj_
	GD13.5 YOLK SAC					
^b^ *Gbp1*	**guanylate-binding protein 1**	15.1	19.23456	+4.265629	5.00E-05	0.070067
*Stc2*	stanniocalcin 2	28.3	15.36555	3.941627	1.25E-12	8.15E-09
*2610528A11Rik*	RIKEN cDNA 2610528A11 gene	20.3	14.19895	3.827712	7.79E-10	3.05E-06
*Gm853*	predicted gene 853	17.5	9.210799	3.203326	1.74E-07	0.000487
*Igfbp1*	insulin-like growth factor-binding protein 1	248.6	7.475471	2.902165	2.08E-14	4.08E-10
^c^ *Adh7*	alcohol dehydrogenase 7 (class IV), μ or σ polypeptide	41.1	7.286177	2.865162	1.67E-10	8.20E-07
^c^ *Zbtb8b*	zinc-finger and BTB domain-containing 8b	22.7	5.996628	2.584151	2.51E-05	0.037875
*Pkhd1l1*	polycystic kidney and hepatic disease 1-like 1	246.1	4.879078	2.286609	1.09E-12	8.15E-09
*Lancl3*	LanC lantibiotic synthetase component C-like 3 (*bacterial*)	24.0	4.443403	2.151665	2.22E-05	0.037875
*Reln*	Reelin	63.4	4.442197	2.151274	7.02E-05	0.091747
*Egln3*	EGL nine homolog 3 (*C. elegans*)	97.8	4.218064	2.076581	4.61E-09	1.51E-05
^b^ *Hemgn*	**Hemogen**	56.0	3.825722	+1.935732	5.02E-07	0.001095
*Slc30a10*	solute carrier family 30, member 10	38.1	3.592453	+1.844969	2.38E-05	0.037875
*Vldlr*	very-low-density lipoprotein receptor	278.3	3.005814	1.587756	2.19E-07	0.000538
^b^ *Hbb-y*	**hemoglobin Y, beta-like embryonic chain**	19218.6	0.306581	−1.70566	2.09E-06	0.0041
	**GD13.5 PLACENTA**					
^b^ *Hemgn*	**hemogen**	72.1	18.87205	+4.238179	2.15E-06	0.045538
^b^ *Gbp1*	**guanylate-binding protein 1**	51.3	5.689857	2.508392	6.52E-06	^d^ 0.069045
	**GD16.5 LIVER**					
*6030408B16Rik*	RIKEN cDNA 6030408B16 gene	693.3	0.179781	−2.47569	3.07E-06	^d^ 0.061137
	**GD16.5 KIDNEY**					
	NO SIGNIFICANT GENES FOUND					
	**GD16.5 LUNG**					
^b^ *Afp*	**alpha-fetoprotein**	569.1	0.256586	−1.96249	5.30E-06	0.008782
*Ahsg*	alpha-2-HS-glycoprotein	68.3	0.241724	−2.04857	6.85E-07	0.001512
*Pzp*	pregnancy-zone protein	121.7	0.234948	−2.08958	2.53E-06	0.005029
*Serpina1c*	serine (or cysteine) peptidase inhibitor, clade A, member 1C	25.8	0.224163	−2.15738	2.67E-05	0.040878
^b^ *Fgg*	**fibrinogen γ-chain**	125.3	0.210643	−2.24712	1.10E-08	5.47E-05
*Serpina1b*	serine (or cysteine) preptidase inhibitor, clade A, member 1B	40.9	0.210225	−2.24999	4.19E-07	0.001042
*Ttr*	transthyretin	61.2	0.209949	−2.25189	8.56E-08	0.000272
^b^ *Kng1*	**kininogen 1**	41.5	0.196759	−2.34550	9.57E-08	0.000272
^b^ *Fgb*	**fibrinogen β-chain**	96.2	0.195113	−2.35762	1.06E-10	2.11E-06
^b^ *Fga*	**fibrinogen α-chain**	90.3	0.191209	−2.38678	3.55E-10	3.53E-06
*Serpina6*	serine (or cysteine) peptidase inhibitor, clade A, member 6	72.8	0.170657	−2.55083	4.07E-09	2.70E-05
^b^ *Ambp*	α1-microglobulin/bikunin	15.3	0.088155	−3.50381	3.98E-06	0.007188
*Itih3*	inter-α trypsin inhibitor, heavy chain 3	25.2	0.084581	−3.56353	2.61E-08	0.000104
	**GD16.5 HEART**					
	NO SIGNIFICANT GENES FOUND					
	**GD16.5 CEREBELLUM**					
	NO SIGNIFICANT GENES FOUND					

^a^RPKM, “number of Reads-Per-Kilobase-of-transcript-per-Million mapped reads”.

^b^Genes that are erythropoiesis- and hypoxia-related – including those involved in iron transport; these also include all platelet and white-cell types and functions because all are derived from hematopoietic stem cells.

^c^These two genes encode Zn^2+^-containing proteins. ADH7 is an enzyme, and ZBTB8B is a Zn^2+^-finger TF.

^d^Although 31 genes are listed, these two are not statistically significant (*i.e. P* > 0.05 when *P*_adj_ is taken into account).

In order to get sufficient numbers of differentially-expressed genes, a relaxed cut-off was used (significant at *P* < 0.05 and absolute fold-change >2) for all statistical functional enrichment analyses. KEGG pathways affected by differentially-expressed genes comprised: in yolk sac, *two* (“malaria” and “African trypanosomiasis”) down-regulated in *Slc39a8(neo/neo)* compared with wild-type; in kidney *one* down-regulated (“tight junction”); and in lung *two* down-regulated (“complement” and “coagulation cascades”) and *one* up-regulated (“*Staphylococcus aureus* infection”). Significant KEGG categories included “complement”, “response to infection”, and “coagulation cascade”. All these pathways are consistent with ZIP8 deficiency causing dysregulated hematopoietic stem cell fate in *Slc39a8(neo/neo)*.

### Global Transcriptome Analysis

**Figure 2:**
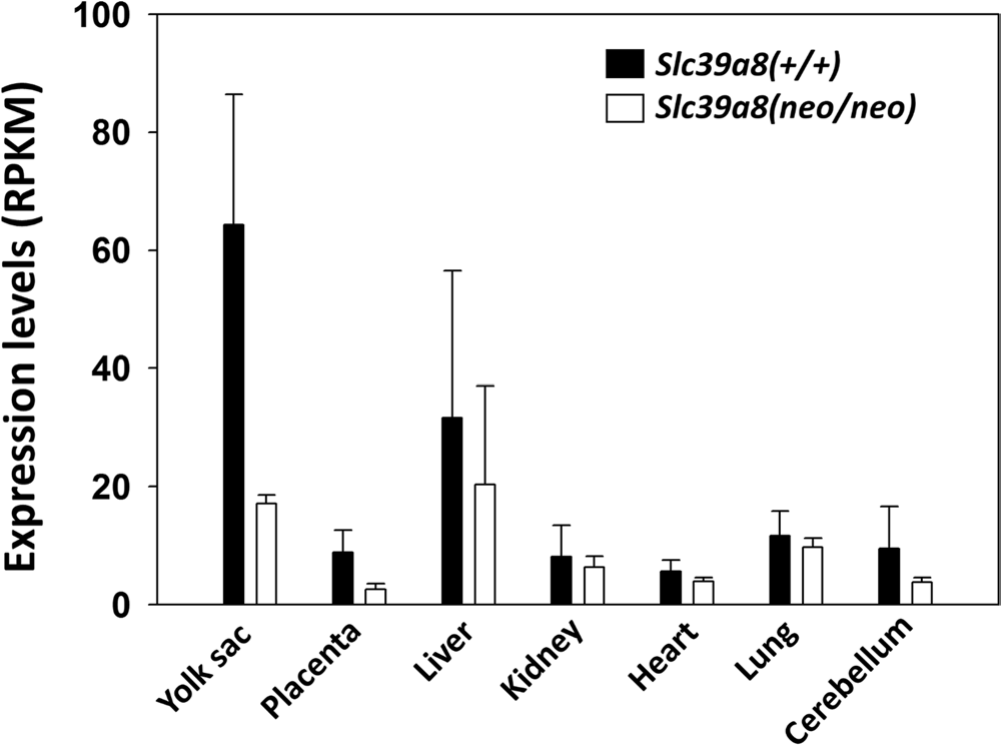
Characterization of *Slc39a8*(+/+) wild-type vs *Slc39a8(neo/neo)* mRNA expression levels in the seven tissues studied. Expression levels are indicated by RPKM, or “number of **R**eads-**P**er-**K**ilobase-of-transcript-per-**M**illion mapped reads.” This analysis takes into account the number of reads, normalized to size of the library, and transcriptional length of each gene. In all tissues, expression levels in *Slc39a8(neo/neo)* samples were lower than those in *Slc39a8*(+/+) wild-type. *Bars* represent average mean values of three determinations, and *brackets* denote S.E.M.

**Figure 3:**
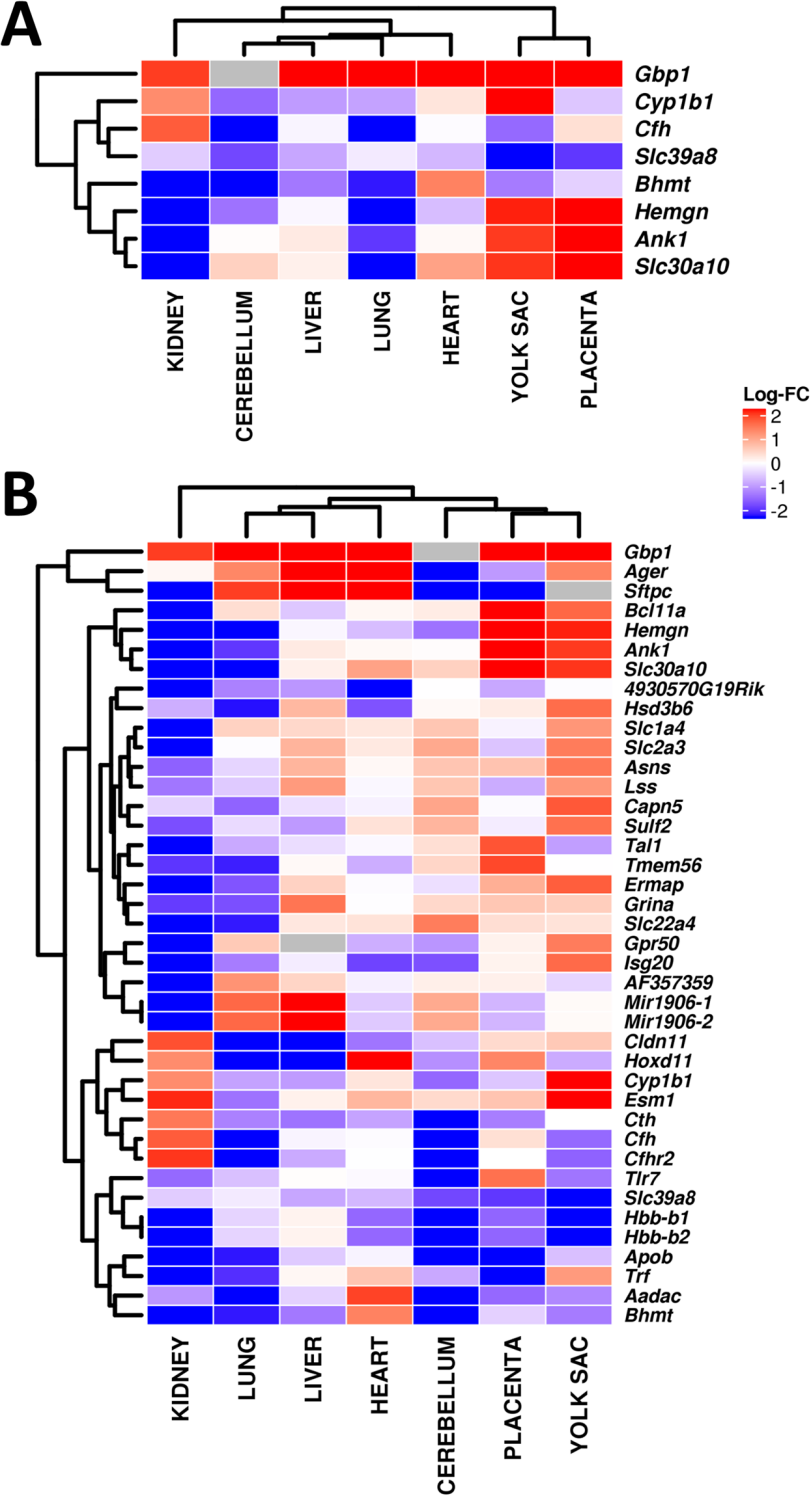
Heat maps plotting log_2_ fold-changes (FC) in differential expression. Using a cut-off of *P* < 0.05, and requiring absolute fold-change of >2.0, the *Slc39a8* gene is also included. (**A**) Differentially-expressed genes in at least three tissues. (**B**) Differentially-expressed genes in at least two tissues. Two hemoglobin genes were significantly differentially down-regulated in all tissues except liver.

RNA-seq analysis confirmed that expression intensities are distinctly different across the seven tissues examined (Fig. 2). Subsequently, global analysis revealed that the number of significantly differentially-expressed genes totaled 695 for all tissues combined (Supplemental Table S1). There were 646 unique genes. *Gbp1* (guanylate-binding protein-1) was found differentially-expressed in five tissues; there were six genes in three tissues (Fig. 3A), and 33 genes in two tissues (Fig. 3B). This resulted in 40 genes (including *Slc39a8*) differentially-expressed in more than one tissue (Supplemental Table S2). The remaining 606 genes were differentially-expressed in only one tissue. Notable among the 40 genes are four *Slc* genes (other than *Slc39a8*) and two hemoglobin genes.

More than 80 differentially-expressed genes (Supplemental Table S1) were associated with hematopoiesis- and hypoxia-related functions (*red font*); these include all myelogenesis functions (*e.g*. cytokines, interferons, innate immunity, and inflammatory functions), because all are derived from hematopoietic stem cells. During mouse in utero development, hematopoiesis transitions from the aorta-gonad-mesonephros (AGM) region and yolk sac (between GD8 and GD13), then to liver (GD11 to GD20), and also to spleen (after GD15.5), and finally to bone marrow after GD17.5. Mouse (but not human) placenta is also a hematopoietic organ^39^.

Many other classes of genes that are seen (Supplemental Table S1) — includes those encoding: Zn^2+^-finger transcription factors (TFs) and homeobox and other very-early-embryonic functions associated with cell cycle and cell division; other Zn^2+^-containing proteins; proteins that are posttranslationally glycosylated; enzymes that participate in lipid and glucose homeostasis; growth factors (including several oncogenes and proto-oncogenes); and G-protein-coupled receptors. Nine cytochrome P450 (*Cyp*) genes, and 27 solute carrier (*Slc*) genes other than *Slc39a8*, were observed; explanations and speculations about members of these two gene superfamilies are discussed in Supplemental Table S1.

### Systematic meta-analysis

**Figure 4:**
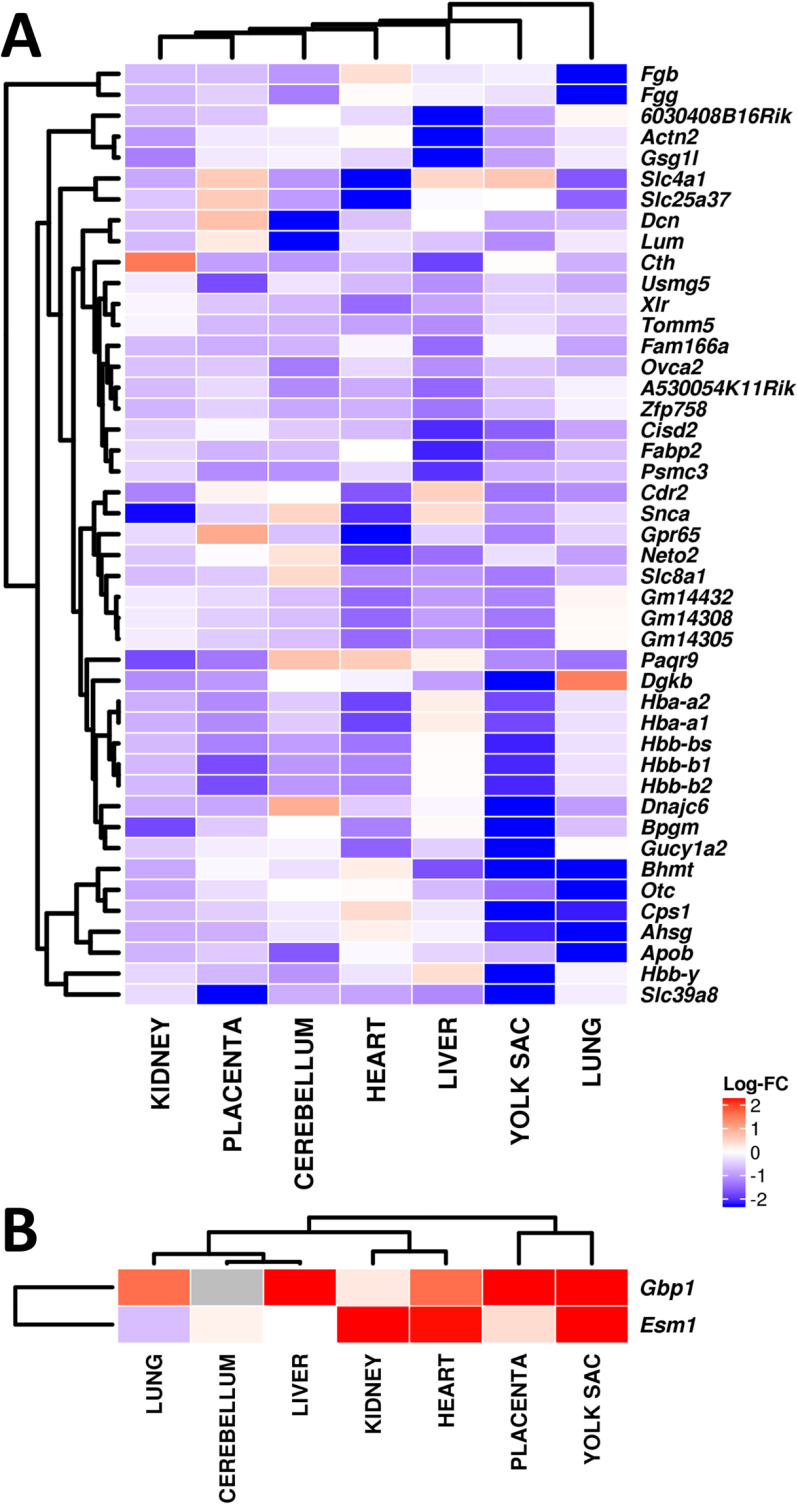
Heat maps, plotting log_2_ fold-changes (FC). A FDR-adjusted meta-*P*-value of <0.1 was used. (**A**) Forty-five differentially-expressed genes that are consistently down-regulated in all tissues. (**B**) The two differentially-expressed genes that are consistently up-regulated in all tissues.

Assuming each tissue is independent from all other tissues, we searched for differentially-expressed genes that tend to change in the same direction in all tissues. A meta-analysis based on Fisher’s combined probability test was applied to integrate empirical *P*-values across all tissues. Forty-seven genes tended to be consistently up- or down-regulated in all tissues (Supplemental Table S3). Not surprisingly, *Slc39a8* was the most consistently down-regulated gene in all seven tissues with a combined *P*-value of 3.5e–04 after FDR adjustment. Six hemoglobin genes were also significantly down-regulated. Heat maps illustrate the 45 down-regulated genes in all tissues (Fig. 4A), and the two up-regulated in all tissues (Fig. 4B).

Among the statistically significant 45 genes being down-regulated, significantly enriched GO categories included: [a] “oxygen carrier activity” (GO:0005344) with *Hbb-b1*, *Hbb-b2*, and *Hbb-y*; [b] “homeostatic processes” (GO:0042592) with *Ahsg*, *Apob*, *Bpgm*, *Cps1*, *Dcn*, *Gpr65*, *Hba-a1*, *Hba-a2, Hbb-b1, Hbb-b2, Otc, Slc25a37, Slc39a8, Slc4a1, Slc8a1*, and *Snca*; and [c] “hemoglobin complexes” (GO:0005833) with *Hba-a1, Hba-a2, Hbb-b1, Hbb-b2, Hbb-bs, Hbb-y*. The down-regulated genes in GO categories GO:0005344 and GO:0005833— plus the four hemoglobin genes included in GO:0042592 category — provide further evidence indicating that globally diminished ZIP8-mediated function results in dysregulated essential hematopoiesis, leading to hypoxia.

### Analysis of transcription factor (TF)-binding sites in genes identified by meta-analysis

Meta-analysis assumes there is a common impact of markedly decreased ZIP8 expression, genome-wide, across all tissues. Transcription is the first step in gene expression, and transcription is modulated by interaction of TFs, having their corresponding binding-sites within specific DNA modules of expressed genes.

After removing *Slc39a8* from Pscan and searching for JASPAR TF motifs in promoter regions, and using a Bonferroni-corrected *P*-value of <0.1, among the “consistently down-regulated genes” discovered by meta-analysis (Supplementary Table S3), we found only two significant motifs — the *Tal1*::*Gata1* heterodimer (JASPAR ID: MA0140.1) and *Nfya* (JASPAR ID: MA0060.1). TAL1 and GATA are of critical importance (*vide infra*). NFYA transcription factors participate in open chromatin of many types of stem cells — participating in differentiation, cell-cycle and transcriptional regulators, splicing and signaling factors, fatty acid and glucose homeostasis, and mitochondrial regulators^40^. Searching for JASPAR TF-binding sites, using the same criterion for the two “consistently up-regulated genes” (Supplemental Table S3), we found no significant motifs.

### Analysis of TF-binding sites in individual tissues

#### Yolk Sac

Based on TF profiles in the JASPAR database, we searched in yolk sac for TF-binding sites enriched by Pscan (Table 3). We found nine up- and down-regulated genes having TF-binding sites significantly enriched in promoters: *Plag1*, *Arnt, Hif1a*, *Znf423*, *Klf13*, *Sp1*, *Gata1*, *Gata2*, and *Gata4*. Note that several TF-binding sites are duplicates with different matrix ID numbers. It is noteworthy that – except for *Arnt* and *Hif1a*, associated with the downstream hypoxic response [*reviewed in*^41^] — the remaining seven are Zn^2+^-finger TFs.

**Table 3:** Significant transcription factor (TF)-binding sites in genes having differential-expression at *P* < 0.05 and absolute fold-change >2; TF-binding site enrichment by Pscan^a^.

TF Name^b^	Matrix ID	Z Score	*P*-value	Bonferroni *P*-value
**UP-REGULATED IN YOLK SAC**				
*Plag1*	MA0163.1	5.12465	1.42E-07	9.00E-05
*Arnt::Hif1a*	MA0259.1	5.09355	1.67E-07	0.000105754
*Znf423*	MA0116.1	4.17542	1.43E-05	0.009050338
*Klf13*	MA0657.1	4.16993	1.45E-05	0.009184069
*Sp1*	MA0079.3	4.04080	2.60E-05	0.016508603
*Glis3*	MA0737.1	3.69324	0.000107782	0.06844157^c^
**DOWN-REGULATED IN YOLK SAC**				
*Gata4*	MA0482.1	4.80921	7.39E-07	0.000469005
*Gata1*	MA0035.3	4.57615	2.31E-06	0.001464596
*Gata1*	MA0035.2	4.12192	1.84E-05	0.011684635
*Gata2*	MA0036.2	3.84142	5.99E-05	0.03805663
**UP-REGULATED IN KIDNEY**				
*Prdm1*	MA0508.1	3.81093	6.77507E-05	0.04302169
**DOWN-REGULATED IN LUNG**				
*Hnf4a*	MA0114.2	5.94132	1.30E-09	8.25E-07
*Hnf4g*	MA0484.1	5.47657	2.01E-08	1.28E-05
*Foxa2*	MA0047.1	5.37203	3.64E-08	2.31E-05
*Hnf4a*	MA0114.3	5.29283	5.63E-08	3.57E-05
*Hnf1b*	MA0153.1	5.20468	9.09E-08	5.77E-05
*Hnf1a*	MA0046.1	4.93196	3.75E-07	0.000238357
*Foxa1*	MA0148.1	4.85155	5.82E-07	0.000369529
*Foxa1*	MA0148.2	4.83018	6.48E-07	0.000411578
*Hnf1b*	MA0153.2	4.7235	1.09E-06	0.000694766
*Hnf4a*	MA0114.1	4.64095	1.64E-06	0.001038854
*Hnf1a*	MA0046.2	4.51127	3.06E-06	0.00194136
*Cux1*	MA0754.1	4.01251	2.88E-05	0.018313464
*Cux2*	MA0755.1	3.9537	3.70E-05	0.02347703
*Foxa2*	MA0047.2	3.85396	5.64E-05	0.035813048
*Foxa1*	MA0148.3	3.72288	9.58E-05	0.060801885^c^
*Cebpe*	MA0837.1	3.677	0.00011437	0.072621775^c^
*Bhlha15*	MA0607.1	3.60496	0.00015141	0.09614281^c^
*Rxrg*	MA0856.1	3.60157	0.00015358	0.097521395^c^
**UP-REGULATED IN HEART**				
*Ebf1*	MA0154.2	3.90828	4.56688E-05	0.02899969

^a^Using this rigorous method of analysis, we found no significant TF-binding sites in placenta, liver or cerebellum.

^b^TF name denotes the TF-binding site(s) enriched by Pscan.

^c^Although 30 enriched TF-binding sites are listed, these five are not statistically significant (*i.e. P* > 0.05 when the Bonferroni-corrected *P*-value is taken into account).

Pleomorphic adenoma gene-1 (*Plag1*) is one of three members in the PLAG family and is best known as an oncogene associated with certain cancers, most notably pleiomorphic tumors of salivary gland; PLAG1 participates in cell proliferation by directly regulating numerous target genes, including growth factors^42^. With regard to *Znf423*, *Klf13* and *Sp1* — there are about 515 *Znf* genes, 18 *Klf* genes, and nine *Sp* genes in mammalian genomes [https://www.genenames.org/]. With regard to *Gata1*, *Gata2* and *Gata4* — GATA factors (*vide infra*) are central to hematopoietic stem cell fate [*reviewed in*^43^].

#### Lung

We found 11 significant TF-binding sites in significantly enriched in promoters of up- and down-regulated genes (Table 3): *Hnf4a*, *Hnf4g*, *Foxa2*, *Hnf1b*, *Hnf1a*, *Foxa1*, *Cux1*, *Cux2*, *Cebpe*, *Bhlha15*, and *Rxrg*. Of these 11 genes, the four *Hnf* genes encode Zn^2+^-finger TFs. The hepatocyte nuclear factor (*Hnf*) family comprises four members that contribute to innumerable critical-life processes. Forkhead genes, which previously had been termed *Hnf* genes, are now officially called *Foxa1*, *Foxa2*, *Foxa3* and *Foxm1* [https://www.genenames.org/]. *Cux* genes code for evolutionarily highly conserved homeobox-domain proteins^44^. With regard to *Cebpe*, *Bhlha15* and *Rxrg* — CEBPE is a leucine-zipper TF associated with several myelogenous leukemias^45^, BHLHA15 is a member of the basic-helix-loop-helix (bHLH) family of TFs involved in numerous developmental programs^46^, and RXRG is a member of the nuclear receptor superfamily that also participates in many morphogenesis processes^47^.

#### Kidney

*Prdm1* was the only TF-binding site significantly enriched in promoters of up- and down-regulated genes (Table 3). *Prdm1* acts as a tumor suppressor gene involved in B-cell and T-cell lymphomas^48^; the PRDM family contains 15 members of epigenetic regulators that participate in autoimmune diseases and infections^49^.

#### Heart

*Ebf1* was the only TF-binding site significantly enriched in promoters of up- and down-regulated genes (Table 3). EBF1 is regarded as a master regulator during B-cell differentiation and also plays a role in B-cell malignancies^50^.

#### Placenta, liver, and cerebellum

We found no statistically significant TF-binding sites as significantly enriched in promoters of up- and down-regulated genes in these tissues.

## Discussion

Because ZIP8 is a known uptake transporter of Zn^2+^, Mn^2+^ and Fe^2+^ — functions of these divalent cations are briefly reviewed.

### Zinc

Intracellular Zn^2+^ is critical in homeostasis-related signal transduction^51^, cell cycle and proliferation^52^, numerous processes that occur during development and differentiation, and maintenance of many Zn^2+^-requiring functions. In mammals there are >100 Zn^2+^-dependent enzymes^53^ and >2,000 Zn^2+^-containing TFs^54^. An independent analysis estimated there are ~2,800 Zn^2+^-binding proteins, corresponding to ~10% of the human proteome^55^. Because these enzymes and TFs carry out vital critical-life functions throughout development — often exerting cell-specific effects on morphogenesis, growth, and differentiation — an embryo’s ability to uphold Zn^2+^ homeostasis is essential from the single-cell-zygote stage onward^56^. Thus, when *Slc39a8(neo/neo)* is compared with *Slc39a8*(+/+), it is likely that many genes listed in Supplemental Table S1 are differentially-expressed as a direct, or indirect, manifestation of deficient ZIP8-mediated Zn^2+^ transport.

### Manganese

Mn^2+^ is also a potent substrate for ZIP8^7,16^. There are several known defects in ZIP-mediated Mn^2+^ transport [*reviewed in*^57^]. A human *SLC39A8* nonsynonymous variant, resulting in decreased ZIP8 transport, impairs the function of Mn^2+^-dependent enzymes — most notably β1,4-galactosyltransferase, essential for biosynthesis of the carbohydrate moiety of glycoproteins; compromised galactosylation in children causes a disorder with deformed skull, severe seizures, short limbs, profound psychomotor retardation, and hearing loss^19^. Because numerous proteins are posttranslationally glycosylated, many genes listed in Supplemental Table S1 might be differentially-expressed as a reflection of defective ZIP8-mediated Mn^2+^ transport. There are no Mn^2+^-containing TFs.

### Iron

ZIP8, along with its closest evolutionarily-related ZIP14 transporter, is a Fe^2+^-transport protein^5,58^. These data might be consistent with studies in the intact animal^13^ — showing that *Slc39a8(neo/neo)* fetuses exhibit dysregulated hematopoiesis — suggestive of an iron-transport defect. As noted (*vide supra*), Supplemental Table S1, highlighted in *red font*, lists >80 differentially-expressed genes associated with dysregulated hematopoietic stem cell fate and subsequent downstream events including hypoxia, when *Slc39a8(neo/neo)* is compared with wild-type; how many of these genes might reflect the importance of Fe^2+^ transport more so than Zn^2+^ transport, will require further studies. Although there are a few Fe^2+^-containing TFs in prokaryotes^59^, there are none in eukaryotes.

### Emergence of several critical-life genetic networks

By comparing transcriptomes in the present study, specific genetic pathways are noteworthy (*vide infra*). There are two possibilities to explain emergence of these genetic pathways in response to globally deficient ZIP8-mediated transport. *One*, perhaps ZIP8-mediated divalent cation transport might be tissue- or cell type-specific — depending upon ZIP8 levels and the abundance of other redundant transport systems in each particular tissue or cell-type. In other words, it is possible that pathways affected by deficient ZIP8-mediated Zn^2+^ transport might be more significant in lung and kidney, those affected by diminished ZIP8-mediated Mn^2+^ transport might be more prominent in liver, and those affected by deficient ZIP8-mediated Fe^2+^ transport might be more important in hematopoietic tissues. *Two*, it is possible that defective Zn^2+^ transport, primarily in early-embryo yolk sac, might override (*i.e*. occur upstream of) Mn^2+^-dependent and/or Fe^2+^-dependent gene products and their functions. These two possibilities will require further experiments, but the present study would argue in favor of the latter, *i.e*. defective Zn^2+^-finger TFs, primarily in yolk sac, cause irreparable impairment of hematopoietic stem cell fate, which is then manifested — developmentally later — in the five fetal organs studied herein.

### Fundamental Importance of TAL1

*Tal1* was up-regulated in placenta and down-regulated in kidney (Supplementary Table S2). Differentially-expressed *Gata* genes were not seen among the 646 unique genes (Supplementary Table S1); this could reflect normal transcription, but then defective function due to its posttranslational Zn^2+^- and/or Mn^2+^-binding requirements. After searching JASPAR TF motifs in promoter regions, among “consistently down-regulated genes” by meta-analysis (Supplemental Table S3), we found the *Tal1*::*Gata1* heterodimeric complex (JASPAR ID: MA0140.1) as an important TF-binding motif. This finding is strengthened when we saw that TF-binding sites enriched by Pscan included *Gata1*, *Gata2* and *Gata4* in yolk sac (Table 3).

TAL1 is a bHLH TF that plays an important role in hematopoietic stem cell development^60^. GATA TFs are Zn^2+^-finger DNA-binding proteins that participate in various biological processes — including hematopoiesis and T-cell development [*reviewed in ref*.^43^]. Shifts in TAL1 occupancy of TF-binding sites during erythroid differentiation are associated with gene repression (*i.e*. dissociation), and activation (*i.e*. co-occupancy with GATA1); in fact, recruitment by GATA TFs appears to be a stronger determinant of TAL1-binding to chromatin than the canonical E-box binding-site motif ^61^.

In order to validate the TAL1 result from PScan, we sought to perform an independent statistical test. From a list of manually-curated 80 TAL1-downstream targets^62^, we intersected the genes in this list with those in Supplemental Table S1 for all tissues (Supplemental Table S4). TAL1 targets were significantly enriched in yolk sac (Fisher’s exact test *P* = 1.178e–05), placenta (*P* = 4.982e–06), and marginally enriched in lung (*P* = 0.01895). Twelve hematopoiesis-related genes were seen, predominantly in yolk sac and placenta. *Egln3* was the most strongly up-regulated gene in yolk sac; EGLN3 is a bHLH TF that responds to hypoxia by decreasing the production of HIF-regulated angiogenic factors^63^. *Cbfa2t3* was the most dramatically down-regulated gene in placenta; CBFA2T3 is clinically associated with several types of human leukemia. The only gene seen in heart was *Zfpm1*; ZFPM1 is a Zn^2+^-finger TF that (like TAL1) also forms heterodimers with TFs of the GATA family.

Moreover, TAL1 is known to activate *Egln3*, *Hemgn* and *Ank1*^62^, and all were up-regulated in *Slc39a8(neo/neo)* yolk sac. TAL1 represses *Hbb-b2*^62^, which was down-regulated in *Slc39a8(neo/neo)* yolk sac (Supplemental Table S4). These data suggest that TAL1 protein activity might be increased in *Slc39a8(neo/neo)* yolk sac, whereas *Tal1* gene differential expression was not seen. Similarly, in *Slc39a8(neo/neo)* placenta, *Hemgn* and *Ank1* were up-regulated and *Hbb-b2* down-regulated — suggesting that TAL1 protein might also be activated in that tissue.

None of the genes encoding Zn^2+^-finger TFs listed in Table 3 was differentially expressed to a level seen in Supplemental Table S1, i*.e*. these genes all appear to be transcribed somewhat normally. Thus, we suggest that, although transcription of these Zn^2+^-finger genes was within normal limits, only the protein functions of these Zn^2+^-finger TFs are the result of ZIP8 deficiency; in other words, postttranslational incorporation of Zn^2+^, and/or deficient posttranslational glycosylation due to diminished Mn^2+^ levels, did not occur normally.

### Fundamental Importance of GATA

The hematopoietically-expressed GATA family members of Zn^2+^-finger TFs are well known to function as key regulators of blood cell fate; for example, phenotypes of knockout mice — deficient in *Gata1*, *Gata2*, or *Gata3* — suggest that these factors each play critical, but distinctly different, roles in hematopoiesis^64^. *GATA2* mutations are clinically associated with familial myelodysplastic syndrome^65,66^, by affecting monocytes and/or B cells.

In order to validate the GATA1 result from Pscan (Table 3), we used a list of 512 GATA1-downstream target genes^67^; this list includes most of the well-established GATA1-mediated erythroid-specific targets, *e.g*. the *Gata1* gene itself, *Gata2*, the β-globin locus (specifically the locus-control region), *Epor*, *Nfe2*, *Slc4a1*, *Gypa*, *Tal1*, *Lrf*, *Klf1*, *Nrf2*, *Runx1* and *Alas2*. We intersected the genes in this list with those in Supplemental Table S1 for all *Slc39a8(neo/neo)* tissues (Supplemental Table S5). Yolk sac was the tissue with the most enriched number of GATA1 targets: excluding *Slc39a8*, there were 13 differentially-expressed GATA1-downstream target genes out of the 512 total (Fisher’s exact test *P* = 0.001369). These findings provide strong evidence for key involvement of GATA1 in many of the differentially-expressed transcriptomic changes in *Slc39a8(neo/neo)* yolk sac.

We found no enrichment of GATA1 targets among differentially-expressed genes in liver, kidney, heart or cerebellum. These data convincingly indicate that deficient ZIP8-mediated transport affects hematopoiesis-related processes primarily in yolk sac – as opposed to effects in placenta, or developmentally later, in early fetal liver.

### Defective Hematopoiesis Leads to Hypoxia

Throughout our analysis presented herein, dysregulation of hematopoiesis-related stem cell functions is repeatedly described; decreases in hemoglobin content, red blood cell number, and iron uptake would likely elicit a “hypoxia signal” in many cell types – resulting in activation of hypoxic-response downstream-target genes. The striking anemia phenotype, seen in the earliest of visible *Slc39a8(neo/neo)* yolk sac, placenta, and embryos [Fig. 1 *and ref*.^13^], supports these findings.

A list of 87 manually-curated HIF-downstream targets^68^ has been reported; examples include genes involved in fatty acid and glucose homeostasis, glycolysis pathway, cell proliferation, cell migration, and angiogenesis. We intersected the genes in this list with those in Supplemental Table S1 for all *Slc39a8(neo/neo)* tissues (Supplemental Table S6). Fifteen differentially-expressed genes were detected in yolk sac (Fisher’s exact test *P* = 4.206e–15), one in kidney, two in lung, and none in placenta, liver, heart or cerebellum; as expected, all 15 of these were up-regulated (Supplemental Table S6). This independent statistical test confirms our *Hif1a* result from Pscan (Table 3).

In lung, *Abcb1a* was up-regulated; *Cyp2s1* and *Trf* down-regulated. ABCB1A (“P-glycoprotein”) is up-regulated in response to many types of stress, including hypoxia^69^. In the presence of hypoxia, HIF1A/ARNT dimers are known to bind to an aryl hydrocarbon receptor response element (AHRE) upstream of the *Cyp2s1* gene^70^; furthermore, the *Arnt*::*Hif1a* heterodimer TF-binding site enriched by Pscan was found to be up-regulated in yolk sac (Table 3). *Trf* encodes transferrin, which is important in Fe^2+^ transport^71^; defective hematopoiesis resulting in anemia and subsequent hypoxia is the likely cause of down-regulation of *Trf* in lung.

In kidney one HIF-downstream target gene (*Met* up-regulated) was found. *Met*, a proto-oncogene, participates in angiogenesis – which would be stimulated by hypoxia^72^.

*Hif1a* expression itself was found not to be differentially-expressed in any tissue, whereas *Hif3a* was up-regulated in yolk sac (Supplementary Table S1). *Hif1a* and *Hif3a* in mice encode hypoxia-inducible TF subunits that respond to low oxygen (pO_**2**_) levels. Either of these TFs can bind as a heterodimeric complex with constitutively-expressed ARNT (aryl hydrocarbon receptor nuclear translocator), product of the *Arnt* gene.

Members of the Egl-9 family of HIF-inducible TFs (*Egln1*, *Egln2* and *Egln3*) were also differentially-expressed (Supplemental Table S1). All six of these above-mentioned genes are members of the bHLH *per-Arnt-sim* (bHLH/PAS) family of TFs that detect endogenous and exogenous signals; the bHLH/PAS family contains at least 30 members in the human and mouse genomes^41^. In fact, two other bHLH/PAS differentially-expressed genes (*Bhlhe40* & *Bhlhe41*) were found to be up-regulated in yolk sac (Supplemental Table S1) – again supporting the theme of the *Slc39a8(neo/neo)* response to hypoxic stress.

### Ingenuity Pathway Analysis

An independent Ingenuity Pathway Analysis (IPA) for the 165 differentially-expressed genes in yolk sac (Supplementary Table S7) revealed major pathways that were consistent with the data discussed throughout the text. Specifically, the top candidate from “Upstream Regulators Analysis” was the transcription factor HIF1A, and it is predicted to be activated. The top categories from the “Diseases and Bio Functions Analysis” included “Transport of molecule,” “Morphology of head,” “Glycolysis of cell,” “Morphology of nervous system,” and “Familial hemolytic anemia” — all of which confirms the data presented herein — as well as the associations of human *SLC39A8* variants with most if not all of the clinical disorders (*vide supra*). With a FDR-adjusted *P*-value < 0.05 from “Canonical Pathway Analysis,” no significant canonical pathways were found.

## Conclusions

**Figure 5:**
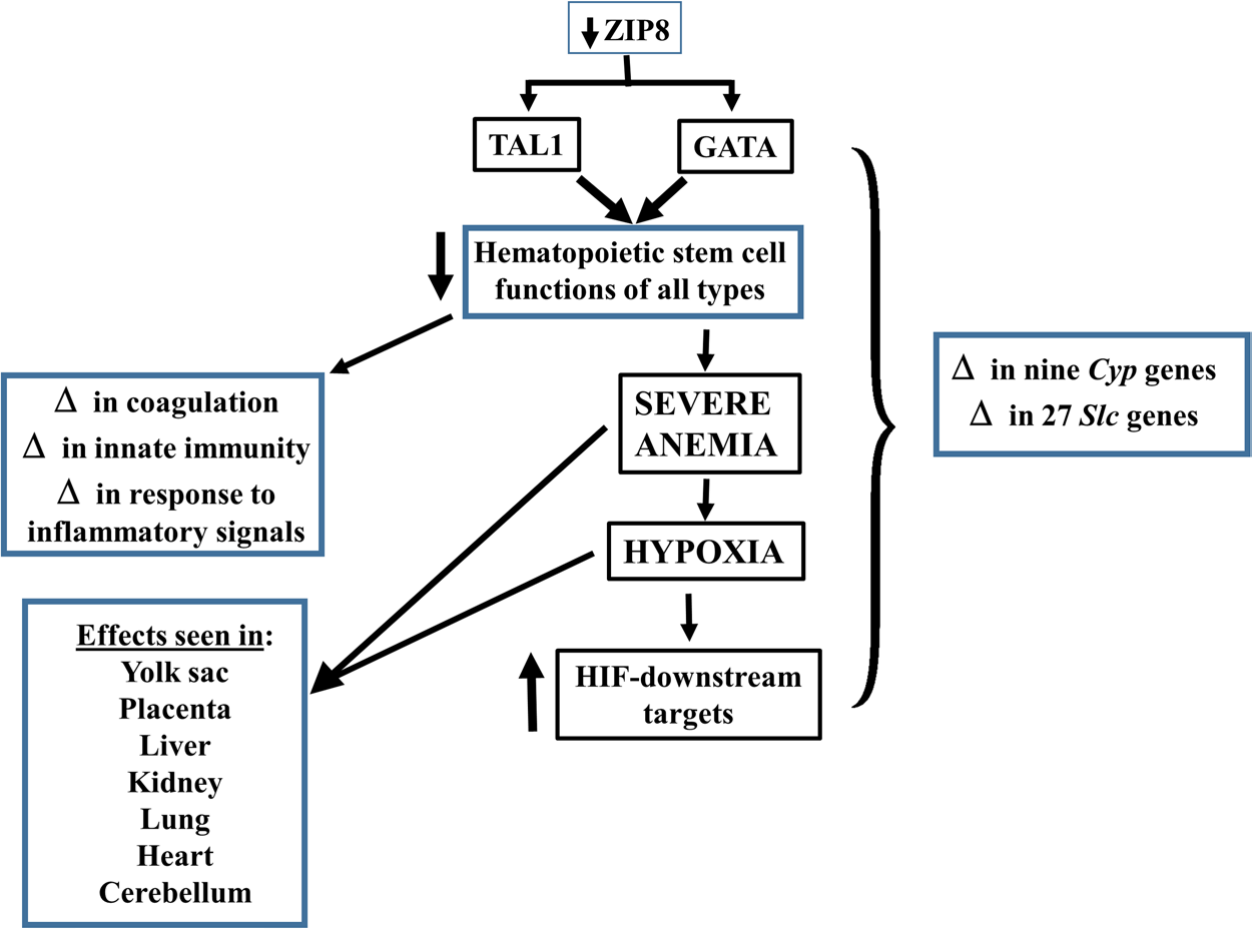
Illustration of critically affected genes and their downstream effects that most closely fit the data presented in the present study. ZIP8 deficiency (*top*), has a major impact on TAL1 and GATA transcription factors (TFs), which appear to function primarily in the hematopoietic stem cells of *Slc39a8(neo/neo)* GD13.5 yolk sac. This function then causes severe dysregulation of hematopoietic stem cell fate and striking anemia in yolk sac, which is visibly obvious [*in* Fig. S2 *of ref*.^13^]. Moreover, hematopoiesis in yolk sac is well known to precede that in liver and then spleen and marrow [*cf*. Fig. S4 *of ref*.^13^]. Downstream effects, as development proceeds, include severe anemia and defects in coagulation, innate immunity, and response to inflammation. The striking anemia leads to a hypoxia response which is seen in all tissues examined, but largely in yolk sac. TAL1, T-cell acute lymphocytic leukemia protein-1 TF. GATA, family of six zinc-finger TFs that regulate hematopoietic stem cell fate. Interactions between TAL1 and GATA exist^61^ [*see text*]. ZIP8-deficiency, plus the result of all these downstream changes, alter the expression of nine *Cyp* genes and 27 *Slc* genes (excluding *Slc39a8*); these changes are mostly unique to one tissue, as detailed in Supplementary Table S1. Δ denotes “changes in”. HIF, hypoxia-inducible factor.

A summary of many of these differentially-expressed genes, and their resultant effects, elicited by ZIP8 deficiency and described throughout this paper, is offered in Fig. 5.

To our knowledge, we know of no studies, reported to date, that dissect the mechanism behind why or how the knockdown of one critically important transporter gene can result in the up- and down-regulation of so many dozens of other transporter genes — and many of the transported moieties are not ions. These intriguing data represent many new avenues of possible experiments for future fascinating studies of whole-genome interactions.

## Material and Methods

### Animals

Cloning details for generating the viable *Slc39a8*(+*/neo)* heterozygous mouse line were described previously^12^. At 6–10 weeks of age, *Slc39a8*(+*/neo)* heterozygous males and females were bred. The morning on which the vaginal plug was found – was considered GD0.5. Individual *Slc39a8*(+/+), *Slc39a8*(+*/neo)* and *Slc39a8(neo/neo)* yolk sacs and placentas at GD13.5 were collected and genotyped; heterozygotes were discarded. Fetal liver, kidney, lung, heart and cerebellum at GD16.5 were also collected and genotyped.

All mouse experiments were conducted in accordance with the National Institutes of Health standards for the care and use of experimental animals. All experimental protocols were approved by the University of Cincinnati College of Medicine (IACUC) Institutional Animal Care and Use Committee [protocol #11-09-12-01].

### Total RNA Extraction

RNeasy Micro kit (Qiagen) was used for total RNA extraction. Each sample, together with Buffer RLT, were added to ceramic-beads (1.4-mm) in tubes and homogenized by using the Precellys homogenizer (Cayman Chemical Company; Ann Arbor, MI). RNA extraction was then performed, following manufacturer’s recommendations.

### RNA-Seq for differential gene expression profiling

For each RNA-seq sample, 1 μg RNA was used (which required two or more mice per sample). RNA-seq was performed at the Genomics, Epigenomics and Sequencing Core at the University of Cincinnati. To prepare the RNA library for sequencing, we used TruSeq RNA Library Prep Kit (Illumina; San Diego, CA), according to manufacturer’s instructions. In brief, poly(A+) RNA was purified from total RNA and then fragmented before cDNA synthesis. Double-strand cDNA fragments were then end-repaired and TA-ligated to the sequencing adapter; the specific index was added to the library during the PCR amplification step. After 12 cycles of PCR enrichment, library quality was evaluated by a Bioanalyzer High Sensitivity chip and quantified by Kapa Library Quantification kit (Kapa Biosystem; Wilmington, MA), using the ABI 9700HT real-time PCR system (Thermo Fisher; Waltham, MA). Next, six individually indexed cDNA libraries were pooled in equal amounts for clustering in the cBot system (Illumina). Libraries were clustered onto a flow cell at concentration of 15 pM, using Illumina’s TruSeq SR Cluster Kit v3, and then sequenced for 50 cycles using TruSeq SBS kit on the Illumina HiSeq system. Each sample was expected to generate ~30 million reads.

### Bioinformatics analysis

Sequence reads were aligned to the genome by using the standard Illumina sequence analysis pipeline, which was analyzed by the Statistical Genomics and Systems Biology Core at the University of Cincinnati. Analyses included determinations of: [a] RNA-seq data quality control (QC) results; [b] all gene expression levels for the RNA samples; and [c] significantly differentially-expressed genes between the two groups, *Slc39a8(neo/neo)* vs *Slc39a8*(+/+). Genes with counts per million (CPM) >1 in at least three samples were used for further analysis. Differential expression analysis was performed using DESeq.^73^. The Benjamini–Hochberg procedure^74^ was applied to adjust *P*-values for False Discovery Rate (FDR). Genes with FDR-adjusted *P*-values of <0.1 were considered as significantly differentially-expressed.

In addition to differential-expression analysis for individual tissues, meta-analysis was performed to identify genes that tend to change in the same direction across all tissues. The meta-analysis used Fisher’s combined probability test to integrate empirical *P*-values from all tissues. The empirical *P*-value for gene ***i*** in tissue ***j*** is defined as r_ij_ = rank(s_**ij**_)/N, and s_**ij**_ = sign(f_**ij**_) **×** [−log_**10**_(p_**ij**_)], where f_**ij**_ is the log_**2**_ fold-change of gene ***i*** in tissue ***j***, p_**ij**_ the *P*-value of differential expression of gene ***i*** in tissue ***j***, and N the total number of genes. See footnote of Supplemental Table S2 for a detailed description. The empirical *P*-value, based on gene rank, was less prone to an extreme effect in any individual tissue; thus, it was more robust, when compared with the original *P*-value from differential analysis. FDR-adjusted *P*-values of <0.1 were used as the cut-off for genes that tend to be differentially-expressed in all tissues.

Functional analysis of differentially-expressed genes was further performed. Because too few genes were significant at FDR < 0.1, a less-stringent cut-off *P*-value of <0.05 and absolute fold-change of >2 was used, in order that a sufficient number of differentially-expressed genes could be used for statistical functional enrichment analysis. This would potentially result in a gene list having an increased number of false positives. Therefore, the purpose of the relaxed cut-off was to explore new hypotheses, rather than to derive individual candidate genes for validations. Specifically, MouseMine^75^ was used to identify significantly enriched gene ontology (GO) categories. Signaling-pathway impact analysis (SPIA) methodology^76^ – with number of differentially-expressed (NDE) genes >1; and FDR-corrected global *P*-value for each gene (pGFdr) <0.2 as cutoff] was used to identify significantly impacted Kyoto Encyclopedia of Genes and Genomes (KEGG)-signaling pathways. Ingenuity Pathway Analysis (IPA) was used to create the summary report and network figure.

In order to search for upstream regulators causing the observed changes in transcriptomes, the transcription-factor-binding site (TFBS) enrichment analysis was performed for promoters of differentially-expressed genes, as well as the significant genes from meta-analysis using Pscan^77^. For each TFBS, Pscan calculates a matching score for each promoter and compares the average matching score in a gene list vs those throughout the rest of the genome. If the average score is significantly higher in that gene list than the rest of the genome, then Pscan flags this TFBS as significant for this gene list. In this analysis, TF binding profiles in the JASPAR 2016 CORE database [http://jaspar.genered.net] was used, and promoter regions from −450 to +50 were considered. Significant TFBS genes were selected by Bonferroni corrected *P*-values of <0.1, as recommended by PScan.

All bioinformatics analyses, except for the tools mentioned above, were performed using the statistical computing platform R, and heat maps were plotted using the R package ComplexHeatmap^78^. We have uploaded our *Slc39a8* RNA-seq data onto Gene Expression Omnibus (GEO). The link to our dataset is https://www.ncbi.nlm.nih.gov/geo/query/acc.cgi?acc=GSE111080.

## Electronic supplementary material

Supplementary Data
