# Supplementary material for: In utero gene expression in the Slc39a8(neo/neo) knockdown mouse
Source: Sci Rep. 2018 Jul 16;8:10703. doi: 10.1038/s41598-018-29109-y (PMC6048144; doi:10.1038/s41598-018-29109-y)
Supplement: Supplementary file 1 — Supplementary Data [file 41598_2018_29109_MOESM1_ESM.pdf]

SUPPLEMENTARY DATA ONLINE

In utero gene expression in the *Slc39a8(neo/neo)* knockdown mouse

Jing Chen[1], **Marina Gálvez-Peralta**[2,*], **Xiang Zhang**[2], **Jingyuan Deng**[2,†], **Zijuan Liu**[3], and **Daniel W. Nebert**[2,‡]

[1]Division of Biomedical Informatics, Cincinnati Children's Hospital Medical Center, Cincinnati, Ohio 45229-3039; [2]Department of Environmental Health, University of Cincinnati College of Medicine, Cincinnati, Ohio 45267-0056; and [3]Department of Biological Sciences, Oakland University, Rochester MI 48309, USA

*Present address: *Department of Pharmaceutical Sciences, West Virginia University Medical Center, Morgantown, WV 26506*

†Present address: *Amazon.com, Inc., Seattle WA 98101*

‡To whom correspondence should be addressed: dan.nebert@uc.edu

TABLE S1. Differentially-expressed genes — using a *P*-value <0.05 and absolute fold-change >2 — when comparing *Slc39a8(neo/neo)* knockdown with *Slc39a8(+/+)* wild-type

GD13.5 YOLK SAC					
**Gene symbol**[a]	**Official gene name**	**Average expression (RPKM)**	**Fold-Change**	**log_2 Fold-Change**	***P*-value**
Gm10377	predicted gene 10377	2.1	NA	**+10**	0.038077
Ccdc170	coiled-coil domain containing 170	2.7	NA	10	0.003263
Ghrh	growth hormone releasing hormone	3.3	NA	10	0.001096
Hdac11	histone deacetylase 11	1.7	NA	10	0.027588
Gbp1[b]	guanylate-binding protein 1	15.1	19.23	4.27	5.00E-05
Stc2	stanniocalcin 2	28.3	15.37	3.94	1.25E-12
Ano2	anoctamin 2	2.9	14.37	3.84	0.017375
2610528A11Rik	RIKEN cDNA 2610528A11 gene	20.3	14.2	3.83	7.79E-10
Etnk2	ethanolamine kinase 2	2.5	11.84	3.57	0.039089
Gng4	guanine nucleotide-binding protein (G protein), γ4	2.3	11.35	3.5	0.038245
Gprc5a	G-protein-coupled receptor, family C, group 5, member A	4.1	9.68	3.28	0.007207
Gm853	predicted gene 853	17.5	9.21	3.2	1.74E-07
Sema7a	sema domain, immunoglobulin domain (Ig), and GPI-membrane anchor, 7A	3.3	8.03	3.01	0.027538
Kcna4	potassium voltage-gated channel, shaker-related subfamily, member 4	8.5	7.85	2.97	0.019251
Aox4	aldehyde oxidase 4	18.3	7.75	2.95	0.000224
Efhc2	EF-hand domain (C-terminal) containing 2	33.3	7.52	2.91	0.040932
Igfbp1[c]	insulin-like growth factor-binding protein 1	248.6	7.48	2.9	2.08E-14
Adh7	alcohol dehydrogenase 7 (class IV), mu or sigma polypeptide	41.1	7.29	2.87	1.67E-10
Ercc2	excision-repair cross-complemntng repair deficiency, complemntation group 2	4.8	7.27	2.86	0.009128
Zbtb8b	zinc finger- and BTB domain-containing 8b	22.7	6	2.58	2.51E-05
Bmp6	bone morphogenetic protein 6	15.3	5.71	2.51	0.001397
Aim1l	absent in melanoma 1-like	3.7	5.5	2.46	0.032743
H2-Ab1	histocompatibility 2, class II antigen A, β1	6.1	5.47	2.45	0.008387
Cyp1b1	cytochrome P450, family 1, subfamily b, polypeptide 1	8.5	5.37	2.43	0.03492
Mgarp	mitochondrial-localized glutamic acid-rich protein	5.8	4.96	2.31	0.011293
Pkhd1l1	polycystic kidney and hepatic disease 1-like 1	246.1	4.88	2.29	1.09E-12
Esm1	endothelial cell-specific molecule 1	583.3	4.62	2.21	0.000122
Ntm	Neurotrimin	5.2	4.48	2.16	0.032329
Lancl3	LanC lantibiotic synthetase component C-like 3 (*bacterial*)	24	4.44	2.15	2.22E-05
Reln	Reelin	63.4	4.44	2.15	7.02E-05
Egln3	EGL nine homolog 3 (*C. elegans*)	97.8	4.22	2.08	4.61E-09
Mapt	microtubule-associated protein τ	5.7	4.04	2.01	0.028332
Sp6	*trans*-acting transcription factor 6	4.7	4.02	2.01	0.038992
Col25a1	collagen, type XXV, α1	4.8	4	2.00	0.049798
Hemgn	hemogen	56	3.83	1.94	5.02E-07
Pitpnm2	phosphatidylinositol transfer protein, membrane-associated 2	10.5	3.6	1.85	0.013494
Slc30a10	solute carrier family 30, member 10	38.1	3.59	1.84	2.38E-05
Snap91	synaptosomal-associated protein 91	6.1	3.55	1.83	0.028576
Ank1	ankyrin 1, erythroid	25.8	3.53	1.82	0.0124
Ap2a1	adaptor-related protein complex 2, α1 subunit	11.1	3.49	1.8	0.005272
Ccdc102a	coiled-coil domain-containing 102A	5.9	3.45	1.79	0.049812
Synpo2	synaptopodin 2	63.7	3.29	1.72	0.00697
Cnpy3	canopy 3 homolog (*zebrafish*; *Danio rerio*)	7.3	3.26	1.71	0.028175
Thoc6	THO complex 6 homolog (*Drosophila*)	8	3.25	1.7	0.028794
Ccdc164	coiled-coil domain containing 164	8	3.15	1.66	0.032467
Slc16a3	solute carrier family 16 (monocarboxylic acid transporters), member 3	13.3	3.11	1.64	0.035615
Capn5	calpain 5	66.5	3.07	1.62	0.03789
Kcne3	potassium voltage-gated channel, Isk-related subfamily, gene 3	17.3	3.07	1.62	0.003336
Gm14047	predicted gene 14047	7.7	3.07	1.62	0.031458
Gch1	GTP cyclohydrolase 1	14.7	3.06	1.62	0.005759

Fscn1	fascin homolog 1, actin bundling protein (*Strongylocentrotus purpuratus*)	17	3.06	1.61	0.003526
Vldlr	very low-density lipoprotein receptor	278.3	3.01	1.59	2.19E-07
Fetub	fetuin β	31.1	3	1.59	0.00862
Ermap	erythroblast membrane-associated protein	132.9	2.99	1.58	0.006552
Pde2a	phosphodiesterase 2A, cGMP-stimulated	10.6	2.98	1.58	0.020801
Mthfd2	MTHF dehydrogenase (NAD+ dependent), methenyltetrahydrofolate cyclohydrolase	24.9	2.97	1.57	0.001119
Krt19	keratin 19	100.7	2.87	1.52	0.000943
Bcl11a	B-cell CLL/lymphoma 11A (zinc-finger protein)	16.6	2.84	1.51	0.006931
A930005H10Rik	RIKEN cDNA A930005H10 gene	13.1	2.83	1.5	0.017392
Pcdh20	protocadherin 20	9.5	2.82	1.5	0.048775
Isg20	interferon-stimulated protein	29.7	2.79	1.48	0.011065
Hsd3b6	hydroxy-Δ-5-steroid dehydrogenase, 3β- and steroid Δ-isomerase 6	470.9	2.75	1.46	0.008695
Spry4	sprouty homolog 4 (*Drosophila*)	31.4	2.74	1.45	0.038453
Sulf2	sulfatase 2	28.1	2.68	1.42	0.014406
Stk32b	serine/threonine kinase 32B	19.6	2.68	1.42	0.005446
Igfbp3[c]	insulin-like growth factor-binding protein 3	134.6	2.67	1.41	0.00298
Mmp19	matrix metallopeptidase 19	9	2.61	1.39	0.04751
Stc1	stanniocalcin 1	11.7	2.61	1.38	0.024014
Gdpd2	glycerophosphodiester phosphodiesterase domain-containing 2	10.9	2.6	1.38	0.0356
Asns	asparagine synthetase	70.7	2.56	1.36	0.000127
Slc2a1	solute carrier family 2 (facilitated glucose transporters), member 1	469.5	2.55	1.35	0.000473
Rhag	Rhesus blood group-associated A glycoprotein	60.2	2.52	1.33	0.000338
Slc2a3	solute carrier family 2 (facilitated glucose transporters), member 3	671.1	2.5	1.32	0.005112
Gpr50	G-protein-coupled receptor 50	17.8	2.49	1.32	0.020918
Yif1a	Yip1-interacting factor homolog A (*S. cerevisiae*)	11.3	2.48	1.31	0.04979
Ndnl2	necdin-like 2	12.6	2.47	1.31	0.028845
Adra2b	adrenergic receptor α2b	16.5	2.46	1.3	0.013904
Sdc1	syndecan 1	11.6	2.39	1.26	0.04787
Bhlhe41	basic helix-loop-helix family, member e41	84.6	2.37	1.25	0.010568
Aldoa	aldolase A, fructose-bisphosphate	275.7	2.35	1.24	0.031794
Mvd	mevalonate (diphospho)decarboxylase	25.5	2.34	1.23	0.006026
Mif	macrophage migration inhibitory factor	18.4	2.34	1.23	0.021821
Zfp94	zinc-finger protein 94	12	2.32	1.22	0.041933
Apoc3	apolipoprotein C-III	12.6	2.31	1.21	0.045566
Eno1	enolase 1α, non-neuronal	269.7	2.3	1.2	0.014886
Cdh3	cadherin 3	70	2.26	1.18	0.001717
Hk1	hexokinase 1	26.6	2.25	1.17	0.024595
Map1b	microtubule-associated protein 1B	391	2.23	1.16	0.001862
Tgfb3	transforming growth factor, β3	29.3	2.22	1.15	0.008699
Basp1	brain-abundant, membrane-attached signal protein 1	64.8	2.22	1.15	0.001918
Bhlhe40	basic helix-loop-helix family, member e40	161.5	2.21	1.14	0.006853
Apln	Apelin	71.5	2.18	1.12	0.011892
Gm5506	predicted gene 5506	483.6	2.17	1.12	0.024416
Plekha2	pleckstrin homol. domain-containing, family A (IP_3-binding specific), memb 2	52.4	2.16	1.11	0.003497
Pm20d2	peptidase M20 domain-containing 2	15.5	2.15	1.11	0.04351
Myadm	myeloid-associated differentiation marker	2123.3	2.14	1.1	0.037611
Tubb2b	tubulin β, class IIB	19.2	2.14	1.1	0.031801
Dio2	deiodinase, iodothyronine, type II	21.9	2.14	1.1	0.038542
Slc38a1	solute carrier family 38, member 1	353.5	2.13	1.09	0.0003
H2-K1	histocompatibility 2, K1, K region	156.7	2.13	1.09	0.00061
Ak4	adenylate kinase 4	326.9	2.13	1.09	0.000395
Slc1a4	solute carrier family 1 (glutamate/neutral amino acid transporters), member 4	47.9	2.12	1.08	0.004676
Lss	lanosterol synthase	404	2.09	1.07	0.00208
Psca	prostate stem-cell antigen	26.5	2.09	1.07	0.018211
Hmgcl	3-hydroxy-3-methylglutaryl-Coenzyme A lyase	26.5	2.09	1.07	0.024636
Ppp1r9b	protein phosphatase 1, regulatory subunit 9B	34	2.09	1.06	0.048548
Lgals3	lectin, galactose-binding, soluble 3	21.8	2.08	1.05	0.049121

Pdgfa	platelet-derived growth factor α	50.3	2.07	1.05	0.007183
Pkm	pyruvate kinase, muscle	371.6	2.06	1.05	0.003951
8430408G22Rik	RIKEN cDNA 8430408G22 gene	113.9	2.06	1.04	0.002176
Prelid2	PRELI domain-containing 2	54.1	2.06	1.04	0.005484
Cited2	Cbp/p300-interacting transactivator, Glu/Asp-rich COOH-terminal domain, 2	315.1	2.06	1.04	0.000622
Atp5d	ATP synthase, H+ transporting, mito F1 complex, Δ subunit	25.1	2.06	1.04	0.025065
Trf	transferrin	19653.8	2.05	1.04	0.013549
Hif3a	hypoxia-inducible factor 3, α subunit	924.9	2.04	1.03	0.000368
Cryl1	crystallin, λ1	27.1	2.04	1.03	0.029265
Map7d1	MAP7 domain-containing 1	25.4	2.03	1.02	0.039494
Stambp	STAM-binding protein	27.2	2.03	1.02	0.023061
Hsd3b2	hydroxy-Δ-5-steroid dehydrogenase, 3β- and steroid Δ-isomerase 2	138.3	2.02	1.02	0.018028
Mt2	metallothionein 2	346.4	2.02	1.02	0.000935
Gpr116	G protein-coupled receptor 116	191.8	2.02	1.02	0.00775
Hunk	hormonally-upregulated Neu-associated kinase	70.7	2.01	1.01	0.016569
Eda2r	ectodysplasin A2 receptor	55.6	0.5	–1.00	0.021863
4932411N23Rik	RIKEN cDNA 4932411N23 gene	99.7	0.5	-1.01	0.005988
Aadac	arylacetamide deacetylase (esterase)	210.9	0.49	-1.03	0.031819
Rimklb	ribosomal-modification protein rimK-like family member B	148.3	0.48	-1.06	0.001323
Prodh2	proline dehydrogenase (oxidase) 2	19.4	0.48	-1.07	0.028371
Dnajc6	DnaJ (Hsp40) homolog, subfamily C, member 6	122.7	0.47	-1.07	0.001134
Hba-a1	hemoglobin alpha, adult chain 1	2545.6	0.46	–1.11	0.026408
Hba-a2	hemoglobin alpha, adult chain 2	2546	0.46	–1.11	0.026455
Slc8a3	solute carrier family 8 (sodium/calcium exchangers), member 3	26.4	0.46	–1.11	0.014213
C030030A07Rik	RIKEN cDNA C030030A07 gene	28	0.46	-1.13	0.01204
Bhmt	betaine-homocysteine methyltransferase	70.7	0.45	-1.14	0.000949
Clec4a3	C-type lectin domain family 4, member a3	13.8	0.44	-1.18	0.042468
Fam220a	family with sequence similarity 220, member A	16.7	0.44	-1.18	0.025718
Marcksl1-ps4	MARCKS-like 1, pseudogene 4	28.5	0.44	-1.19	0.007226
Tlr7	toll-like receptor 7	20.7	0.44	-1.2	0.013775
Aadat	aminoadipate aminotransferase	31.3	0.43	-1.22	0.005303
1810062O18Rik	RIKEN cDNA 1810062O18 gene	10.8	0.43	-1.22	0.04315
Cfh	complement component factor h	203	0.41	-1.3	0.014087
Pxmp2	peroxisomal-membrane protein 2	8.7	0.35	-1.51	0.03088
Magea5	melanoma antigen, family A, 5	6.3	0.33	-1.61	0.049439
Slc6a15	solute carrier family 6 (neurotransmitter transporters), member 15	7.2	0.32	–1.65	0.037471
Grm7	glutamate receptor, metabotropic 7	6.2	0.31	-1.69	0.035742
Clec5a	C-type lectin-domain family 5, member a	5.8	0.31	-1.7	0.04088
Slc5a11	solute carrier family 5 (sodium/glucose cotransporters), member 11	5.7	0.31	–1.70	0.041006
Hbb-y	hemoglobin Y, β-like embryonic chain	19218.6	0.31	–1.71	2.09E-06
Gm101	predicted gene 101	7.2	0.31	-1.71	0.019502
BC049635	cDNA sequence BC049635	6.1	0.29	-1.78	0.035888
Esrrb	estrogen-related receptor β	9.4	0.28	-1.85	0.005738
Gm6642	ubiquitin-like 5 pseudogene	6.6	0.28	-1.85	0.033137
Vmn2r12	vomeronasal 2, receptor 12	8	0.27	-1.91	0.011006
Gabra1	γ-aminobutyric acid (GABA) A receptor, subunit α1	14.5	0.27	-1.91	0.00112
2900002K06Rik	RIKEN cDNA 2900002K06 gene	6	0.25	-1.99	0.025151
Elavl2	ELAV (embryonic lethal, abnormal vision, *Drosophila*)-like 2 (Hu antigen B)	6.2	0.25	-2.01	0.019532
Slc39a8	solute carrier family 39 (metal ion transporters), member 8	981	0.25	–2.02	0.003534
Dgkb	diacylglycerol kinase, β	17.3	0.24	-2.04	0.003267
Hbb-b1	hemoglobin, beta adult major chain	7608.1	0.21	–2.23	0.01473
Hbb-b2	hemoglobin, beta adult minor chain	7608.1	0.21	–2.23	0.01473
Beta-s	hemoglobin subunit β1-like	6346.6	0.21	–2.27	0.01333
Olfr857	olfactory receptor 857	11.4	0.14	-2.79	0.020188
Ifi202b	interferon-activated gene 202B	20.7	0.04	-4.48	0.006328
Kdm5d	lysine (K)-specific demethylase 5D	213	NA	-10	0.033431
Gpr111	G protein-coupled receptor 111	1.3	NA	-10	0.048708
Ccdc81	coiled-coil domain-containing 81	1.5	NA	-10	0.030022

GD13.5 PLACENTA					
**Gene symbol**	**Official gene name**	**Average expression (RPKM)**	**Fold-Change**	**log_2 Fold-Change**	***P*-value**
Slc43a1	solute carrier family 43, member 1	2.6	21.8	+4.45	0.029887
Hemgn	Hemogen	72.1	18.87	4.24	2.15E-06
Slc10a6	solute carrier family 10 (sodium/bile acid cotransporter family), member 6	3.4	13.63	3.77	0.030592
Tmem229a	transmembrane protein 229A	2.8	13.5	3.75	0.02237
Gm16617	predicted gene, 16617	3	8.42	3.07	0.0404
Ank1	ankyrin 1, erythroid	5.4	7.76	2.96	0.00739
Vamp1	vesicle-associated membrane protein 1	3.5	7.23	2.85	0.045509
Gbp1	guanylate-binding protein 1	51.3	5.69	2.51	6.52E-06
Fam196b	family with sequence similarity 196, member B	6.8	4.88	2.29	0.019586
D330022K07Rik	RIKEN cDNA D330022K07 gene	230	4.55	2.19	0.007746
Bcl11a	B-cell CLL/lymphoma 11A (zinc-finger protein)	21.5	4.28	2.1	0.000456
Dusp14	dual-specificity phosphatase 14	7.9	4.08	2.03	0.009089
Nxpe2	neurexophilin and PC-esterase domain family, member 2	35.5	3.76	1.91	0.002373
Msl3l2	male-specific lethal 3-like 2 (*Drosophila*)	9.3	3.37	1.75	0.017999
Oit3	oncoprotein-induced transcript 3	9.8	3.29	1.72	0.022152
Tal1	T-cell acute lymphocytic leukemia 1	16.1	3.15	1.65	0.038062
Prl8a2	prolactin family 8, subfamily a, member 2	1908.2	2.97	1.57	0.012757
Gm15713	predicted gene 15713	16.1	2.94	1.55	0.012836
Rgs18	regulator of G-protein signaling 18	30.7	2.85	1.51	0.003008
1500015A07Rik	RIKEN cDNA 1500015A07 gene	12.7	2.77	1.47	0.022345
Slc25a2	solute carrier family 25 (mitochondrial carrier, ornithine transporter) member 2	9.8	2.71	1.44	0.041635
St8sia6	ST8 α-*N*-acetyl-neuraminide α-2,8-sialyltransferase 6	26.1	2.64	1.4	0.03219
Gbp3	guanylate-binding protein 3	53.2	2.48	1.31	0.004881
Malat1	metastasis-associated lung adenocarcinoma transcript 1 (non-coding RNA)	264438.1	2.44	1.29	0.027408
Tmc7	transmembrane channel-like gene family 7	60.8	2.39	1.26	0.007686
Ctsk	cathepsin K	592.6	2.39	1.26	0.04233
Atp8a1	ATPase, aminophospholipid transporter (APLT), class I, type 8A, member 1	262.3	2.37	1.25	0.003137
Cep128	centrosomal protein 128	64.8	2.36	1.24	0.004782
Sulf1	sulfatase 1	25.2	2.34	1.23	0.046307
Mns1	meiosis-specific nuclear structural protein 1	17	2.33	1.22	0.030083
Dhrs9	dehydrogenase/reductase (SDR family) member 9	89	2.33	1.22	0.004546
Usp53	ubiquitin-specific peptidase 53	1441.3	2.29	1.19	0.049738
Rrm2	ribonucleotide reductase M2	464	2.26	1.18	0.023818
Ikzf1	IKAROS family zinc-finger 1	32	2.24	1.17	0.033308
Cped1	cadherin-like and PC-esterase domain-containing 1	104.5	2.21	1.14	0.043615
Trim30d	tripartite motif-containing 30D	57.8	2.18	1.12	0.00915
Lyve1	lymphatic-vessel endothelial hyaluronan receptor 1	135.2	2.17	1.12	0.007705
Gpx8	glutathione peroxidase 8 (putative)	139	2.11	1.08	0.006049
Atp13a2	ATPase type 13A2	78.1	2.08	1.06	0.01376
Adal	adenosine deaminase-like	37.9	2.07	1.05	0.023871
E230016K23Rik	RIKEN cDNA E230016K23 gene	47.9	2.04	1.03	0.041235
Gm14057	Ppp1r14c pseudogene	28.9	2.03	1.02	0.044186
Pet100	PET100 homolog (*S. cerevisiae*)	104.4	0.5	–1.01	0.010124
Pld5	phospholipase D family, member 5	31.2	0.5	-1.01	0.043083
Cdh1	cadherin 1	331.7	0.49	-1.02	0.007064
Pde6a	phosphodiesterase 6A, cGMP-specific, rod, α	36	0.49	-1.03	0.033413
Rpl41	ribosomal protein L41	976.6	0.48	-1.07	0.028875
Rpl19	ribosomal protein L19	134.4	0.48	-1.07	0.024411
Fau	Finkel-Biskis-Reilly murine sarcoma virus (FBR-MuSV) ubiquitously-expressd	311.8	0.48	-1.07	0.017805
1500004A13Rik	RIKEN cDNA 1500004A13 gene	56.7	0.47	-1.08	0.036297
Rps16	ribosomal protein S16	69.3	0.47	-1.1	0.026093
Pskh1	protein serine kinase H1	37.4	0.46	-1.13	0.017623
Rps15	ribosomal-protein S15	121.1	0.45	-1.15	0.01362
Gjb5	gap-junction protein, β5	33.3	0.42	-1.24	0.009968
Epn3	epsin 3 (target of *Trp53*)	40	0.42	-1.26	0.004879

Hbb-b1	hemoglobin, beta adult major chain	1242.6	0.4	–1.34	0.028901
Hbb-b2	hemoglobin, beta adult minor chain	1242.6	0.4	–1.34	0.028901
Rps11	ribosomal-protein S11	95	0.39	-1.35	0.009822
Smarca1	SWI/SNF-related, matrix-associated, actin-dependent regulator of chromatin, subfamily a, member 1	82.8	0.39	-1.37	0.017411
Maz	MYC-associated zinc-finger protein (purine-binding transcription factor)	18.8	0.38	-1.38	0.046655
Ano3	anoctamin 3	10.2	0.38	-1.39	0.04212
Tubb3	tubulin, β3 class III	13.5	0.38	-1.4	0.021319
Pla2g12a	phospholipase A_2, group XIIA	17.2	0.38	-1.41	0.03545
Col11a1	collagen, type XI, α1	78.4	0.38	-1.41	0.03266
Rab9b	RAB9B, member RAS oncogene family	8.9	0.35	-1.52	0.047809
Csf1	colony-stimulating factor 1 (macrophage)	20.8	0.34	-1.55	0.004804
A430089I19Rik	RIKEN cDNA A430089I19 gene	8	0.34	-1.56	0.04584
Lrsam1	Leu-rich repeat and sterile α-motif-containing 1	10.3	0.34	-1.56	0.02876
Slc39a8	solute carrier family 39 (metal ion transporter), member 8	109	0.31	–1.71	1.94E-05
Mrps28	mitochondrial-ribosomal protein S28	6.6	0.3	-1.74	0.045011
Gaa	glucosidase, α, acid	8.4	0.3	-1.76	0.027864
Efnb1	ephrin B1	8.8	0.29	-1.76	0.016407
2810001G20Rik	RIKEN cDNA 2810001G20 gene	6.6	0.28	-1.85	0.035636
Ppargc1a	peroxisome proliferative-activated receptor-γ, coactivator 1α	23	0.27	-1.91	0.002026
Gm21637	predicted gene, 21637	5.2	0.24	-2.04	0.036323
Gm14354	predicted gene 14354	5.2	0.24	-2.04	0.036323
Ifitm10	interferon-induced transmembrane protein 10	5.3	0.23	-2.13	0.035472
Ict1	immature colon carcinoma transcript 1	5.2	0.22	-2.18	0.031189
Rmrp	RNA component of mitochondrial RNAase P	11.2	0.22	-2.18	0.034409
Prol1	proline rich, lacrimal 1	11.8	0.21	-2.22	0.048119
Cacna1e	calcium channel, voltage-dependent, R type, α 1E subunit	4	0.19	-2.38	0.045875
Gm6150	predicted gene 6150	4.2	0.19	-2.43	0.031557
Rpph1	ribonuclease P RNA component H1	44	0.18	-2.47	0.021699
Gm15023	predicted gene 15023	4.4	0.17	-2.6	0.021963
AV320801	expressed sequence AV320801	4.4	0.17	-2.6	0.021963
Neurog2	neurogenin 2	48.6	0.14	-2.85	2.63E-05
Nudt11	nudix (nucleoside diphosphate linked moiety X)-type motif 11	4.2	0.13	-2.98	0.012352
Gpr81	G-protein-coupled receptor 81	2.3	0.09	-3.55	0.046167
Olfml2b	olfactomedin-like 2B	2.4	0.07	-3.78	0.049678
Trhde	TRH-degrading enzyme	2.5	0.07	-3.83	0.02367
Acot4	acyl-CoA thioesterase 4	2.5	0.06	-3.99	0.023963
Tacr3	tachykinin receptor 3	3.8	0.05	-4.47	0.029893
Cbfa2t3	core-binding factor, runt domain, α-subunit 2, translocated to, 3 (human)	4	0.04	-4.55	0.006988
Rps19bp1	ribosomal-protein S19-binding protein 1	1.6	NA	-10	0.047475

GD16.5 LIVER

**Gene symbol**	**Official gene name**	**Average expression (RPKM)**	**Fold-Change**	**log_2 Fold-Change**	***P*-value**
Anks1	ankyrin repeat and SAM domain-containing 1	3.8	NA	**+10**	0.034196
B430319G15Rik	RIKEN cDNA B430319G15 gene	8.7	6.42	2.68	0.041893
Gbp1	guanylate-binding protein 1	23.5	6.35	2.67	0.003716
Mir1906-1	microRNA 1906-1	10.5	5.43	2.44	0.046248
Mir1906-2	microRNA 1906-2	10.5	5.43	2.44	0.046248
Epor	erythropoietin receptor	18.5	3.66	1.87	0.035935
Slc6a9	solute carrier family 6 (neurotransmitter transporter, glycine), member 9	53.4	3.1	1.63	0.021275
Frat2	frequently-rearranged in advanced T-cell lymphomas 2	40.3	2.72	1.44	0.041021
Lars2	leucyl-tRNA synthetase, mitochondrial	4976.1	2.69	1.43	0.006643
Grina	glutamate receptor, ionotropic, N-CH_3-D-Asp-associated protein 1 (Glx-binding)	60	2.62	1.39	0.042098
Ip6k1	inositol hexaphosphate kinase 1	116.7	2.37	1.25	0.037276
Acer2	alkaline ceramidase 2	77.5	2.33	1.22	0.037935
Fads1	fatty-acid desaturase 1	555.8	2.3	1.2	0.022541
Glipr2	GLI pathogenesis-related 2	132.8	2.21	1.14	0.04476

Supt4a	suppressor of Ty 4A	345.5	2.18	1.13	0.049975
Garem	GRB2-associated, regulator of MAPK1	185.8	2.14	1.1	0.042327
Pdcd5	programmed cell death 5	777.1	0.45	-1.15	0.023395
Bhmt	betaine-homocysteine methyltransferase	964.9	0.44	-1.17	0.030857
Cyp3a11	**cytochrome P450, family 3, subfamily a, polypeptide 11**	505.2	0.43	–1.20	0.029067
Cth	cystathionase (cystathionine γ-lyase)	451.3	0.43	-1.22	0.023948
Cisd2	CDGSH iron-sulfur domain 2	489.5	0.43	-1.22	0.015569
2810004N23Rik	RIKEN cDNA 2810004N23 gene	199.3	0.42	-1.24	0.046931
Psmc3	proteasome (prosome, macropain) 26S subunit, ATPase 3	362.8	0.42	-1.26	0.017579
Pon1	paraoxonase 1	88.4	0.41	-1.28	0.01868
Fabp2	fatty-acid-binding protein 2, intestinal	254.9	0.4	-1.33	0.013452
Gm3500	predicted gene 3500	78.5	0.4	-1.34	0.036891
Med21	mediator complex subunit 21	165.8	0.39	-1.36	0.01525
Gm3696	predicted gene 3696	43.1	0.38	-1.38	0.045917
Chpt1	choline phosphotransferase 1	294.9	0.37	-1.43	0.00517
Dcaf12l1	DDB1- and CUL4-associated factor 12-like 1	36.6	0.33	-1.59	0.031118
Ugt3a1	UDP glycosyltransferase 3 family, polypeptide A1	41.4	0.31	-1.68	0.009639
Actn2	actinin α2	1294.9	0.31	-1.7	0.000942
Gsg1l	GSG1-like	451.6	0.3	-1.74	0.001029
Ces1c	carboxylesterase 1C	67.7	0.25	-1.98	0.031081
Car3	carbonic anhydrase 3	442.3	0.23	-2.14	0.013403
Eya1	eyes absent 1 homolog (*Drosophila*)	12.1	0.21	-2.27	0.025619
Pcdh15	protocadherin 15	13	0.2	-2.32	0.025444
Ugt3a2	UDP glycosyltransferase 3 family, polypeptide A2	70.8	0.19	-2.37	0.004476
6030408B16Rik	RIKEN cDNA 6030408B16 gene	693.3	0.18	-2.48	3.07E-06
Gm10413	predicted gene 10413	8.9	0.13	-2.9	0.030739
Gm5795	predicted gene 5795	8.9	0.13	-2.9	0.030739
Fam13c	family with sequence similarity 13, member C	5.9	0.08	-3.66	0.032405
Phyhipl	phytanoyl-CoA hydroxylase-interacting protein-like	4.5	0.04	-4.63	0.048457
Col6a5	collagen, type VI, α5	5.6	0.03	-4.96	0.016125
Mup15	major urinary protein 15	11.9	NA	-10	0.035407
Gm15085	predicted gene 15085	5.5	NA	-10	0.004892
Lmo3	LIM domain only 3	3.7	NA	-10	0.02402
Mep1a	meprin 1α	3.1	NA	-10	0.044104
Mup2	major urinary protein 2	21.6	NA	-10	0.042211
Ott	ovary testis transcribed	5.5	NA	-10	0.004892
Slc5a1	solute carrier family 5 (Na/glucose cotransporter), member 1	6.8	NA	–10.00	0.024828
Abca16	ATP-binding cassette, sub-family A (ABC1), member 16	3.4	NA	-10	0.038952
Gm6367	predicted gene 6367	3.4	NA	-10	0.038952
Clca6	chloride channel calcium-activated 6	3.8	NA	-10	0.022852
**GD16.5 KIDNEY**					
**Gene symbol**	**Official gene name**	**Average expression (RPKM)**	**Fold-Change**	**$\log_2$ Fold-Change**	***P*-value**
Fbp1	fructose bisphosphatase 1	8.9	7.39	**+2.88**	0.043426
Defb1	defensin β1	9.9	6.68	2.74	0.041522
Slc22a19	solute carrier family 22 (organic anion transporter), member 19	28.1	6.65	2.73	0.013501
Cndp1	carnosine dipeptidase 1 (metallopeptidase M20 family)	19.8	5.68	2.51	0.018248
Ppp1r42	protein phosphatase 1, regulatory subunit 42	17	5.25	2.39	0.027867
Gsdmc3	gasdermin C3	83.5	5.23	2.39	0.01128
Mcoln3	mucolipin 3	19.8	5.07	2.34	0.010291
Gsdmc4	gasdermin C4	34.5	4.7	2.23	0.019218
Abca13	ATP-binding cassette, sub-family A (ABC1), member 13	133.2	4.59	2.2	0.010566
Gsdmc2	gasdermin C2	87.6	4.55	2.19	0.011279
Slc12a1	solute carrier family 12, member 1	970.6	4.14	2.05	0.002836
Cldn16	claudin 16	16.6	3.99	2	0.034985
Col4a4	collagen, type IV, α4	27.8	3.86	1.95	0.018653
Esm1	endothelial cell-specific molecule 1	151.8	3.74	1.9	0.001607
Pkhd1	polycystic kidney and hepatic disease 1	569.6	3.72	1.9	0.001486
4930442L01Rik	RIKEN cDNA 4930442L01 gene	20.2	3.7	1.89	0.037006
1700109F18Rik	RIKEN cDNA 1700109F18 gene	45.3	3.6	1.85	0.011631
Cfhr2	complement factor H-related 2	532.7	3.58	1.84	0.001066

Nphs2	nephrosis 2 homolog, podocin (human)	168.4	3.51	1.81	0.004109
Rsad2	radical *S*-adenosyl methionine domain-containing 2	199.9	3.47	1.8	0.002213
Fmo2	flavin-containing monooxygenase 2	43.9	3.4	1.76	0.023056
4930533B01Rik	RIKEN cDNA 4930533B01 gene	23.8	3.39	1.76	0.047078
Sostdc1	sclerostin domain-containing 1	283.9	3.39	1.76	0.001895
Plscr2	phospholipid scramblase 2	75.9	3.37	1.75	0.025821
Mep1b	meprin 1β	62.9	3.37	1.75	0.031021
Cldn11	claudin 11	164.2	3.21	1.68	0.002362
Plau	plasminogen activator, urokinase	91.5	3.18	1.67	0.007402
Cubn	cubilin (intrinsic factor-cobalamin receptor)	422.6	3.12	1.64	0.017264
Neat1	nuclear paraspeckle assembly transcript 1 (non-protein coding)	502.5	3.11	1.64	0.030174
Nkx3-1	NK-3 transcription factor, locus 1 (*Drosophila*)	28.9	3.11	1.64	0.03619
Tinag	tubulointerstitial-nephritis antigen	38.8	3.08	1.62	0.028105
Lrp2	low-density lipoprotein receptor-related protein 2	2651.6	3.05	1.61	0.0124
Cfh	complement component factor h	4429.2	3.02	1.59	0.0028
Cldn8	claudin 8	467.7	3	1.59	0.003548
Btnl9	butyrophilin-like 9	68.1	2.99	1.58	0.02331
Slco1a4	solute carrier organic anion transporter family, member 1a4	51	2.95	1.56	0.029147
Gm4788	predicted gene 4788	441.4	2.9	1.54	0.005177
Ehf	ets homologous factor	492	2.88	1.53	0.004413
Atp6v0a4	ATPase, H+ transporting, lysosomal V0 subunit A4	134.7	2.86	1.52	0.048821
1110028F11Rik	RIKEN cDNA 1110028F11 gene	38.4	2.85	1.51	0.03568
Hoxd3	homeobox D3	120.1	2.82	1.5	0.009016
Npr3[d]	natriuretic-peptide receptor 3	515.9	2.77	1.47	0.007925
Mme	membrane metallo endopeptidase	1285.3	2.73	1.45	0.01965
Samd9l	sterile α-motif domain-containing 9-like	365	2.72	1.44	0.011491
Cyp7b1	**cytochrome P450, family 7, subfamily b, polypeptide 1**	72.8	2.66	1.41	0.024117
AI314831	expressed sequence AI314831	86.8	2.64	1.4	0.019515
D030045P18Rik	RIKEN cDNA D030045P18 gene	53	2.63	1.4	0.048051
Ly6a	lymphocyte antigen 6 complex, locus A	206.3	2.59	1.37	0.017636
Tmem52b	transmembrane-protein 52B	231.5	2.59	1.37	0.014651
Alx1	ALX homeobox 1	107.5	2.58	1.37	0.013814
Nabp1	nucleic acid-binding protein 1	263.4	2.58	1.37	0.024406
Cth	cystathionase (cystathionine γ-lyase)	92.7	2.56	1.36	0.045199
Ace2[e]	angiotensin I-converting enzyme (peptidyl-dipeptidase A) 2	699.3	2.54	1.34	0.012319
Tm4sf1	transmembrane 4 superfamily member 1	178.1	2.51	1.33	0.025193
Slc47a1	solute carrier family 47, member 1	94.2	2.44	1.28	0.038417
Snord22	small nucleolar RNA, C/D box 22	98.5	2.43	1.28	0.042851
Tmprss2	transmembrane protease, serine 2	82.8	2.41	1.27	0.028147
Hoxa10	homeobox A10	180.7	2.4	1.26	0.018985
Eya4	eyes absent 4 homolog (*Drosophila*)	427	2.39	1.26	0.017776
Mir17hg	Mir17 host gene 1 (non-protein coding)	447.5	2.38	1.25	0.02446
Hrsp12	heat-responsive protein 12	380.7	2.37	1.25	0.036323
9130206I24Rik	RIKEN cDNA 9130206I24 gene	63.6	2.36	1.24	0.048052
Ocln	Occludin	101.8	2.35	1.23	0.045698
Hoxd10	homeobox D10	183.3	2.34	1.23	0.021565
BC021891	cDNA sequence BC021891	77	2.34	1.23	0.044348
Mctp2	multiple C2 domains, transmembrane 2	299.4	2.33	1.22	0.02828
Wt1	Wilms tumor 1 homolog	837.5	2.31	1.21	0.026022
Met	met proto-oncogene	517.8	2.3	1.2	0.022918
Ros1	Ros1 proto-oncogene	272	2.28	1.19	0.020944
Rnf128	ring-finger protein 128	259.7	2.26	1.18	0.042698
Cyp1b1	**cytochrome P450, family 1, subfamily b, polypeptide 1**	284.5	2.26	1.17	0.032379
Hoxd11	homeobox D11	664.7	2.25	1.17	0.021077
Epcam	epithelial cell adhesion molecule	409	2.25	1.17	0.020497
Aim1	absent in melanoma 1	176.2	2.25	1.17	0.031708
Hoxd8	homeobox D8	132.7	2.24	1.17	0.043709
Cdc14a	CDC14 cell-division cycle 14A	339.9	2.23	1.16	0.038619
Gimap4	GTPase, IMAP family member 4	616.1	2.23	1.16	0.032168
Leprel1	leprecan-like 1	252.7	2.23	1.16	0.041677
Hmcn1	hemicentin 1	2504.1	2.19	1.13	0.025176
Shcbp1	Shc SH2-domain-binding protein 1	504.2	2.17	1.12	0.04345
Dcdc2a	doublecortin domain-containing 2a	1358.4	2.14	1.10	0.037054

Gm10664	predicted gene 10664	141.5	2.13	1.09	0.036868
Mecom	MDS1 and EVI1 complex locus	1279.5	2.11	1.08	0.035085
Tfcp2l1	transcription factor CP2-like 1	1897.8	2.1	1.07	0.032161
Hoxb7	homeobox B7	273.1	2.08	1.06	0.047805
Heph	hephaestin	374.3	2.07	1.05	0.039407
Calb1	calbindin 1	1117.2	2.07	1.05	0.04244
Frem1	Fras1-related extracellular-matrix protein 1	2090.7	2.05	1.03	0.040591
Ptn	pleiotrophin	1262.8	0.48	–1.05	0.044521
Map4	microtubule-associated protein 4	1191	0.46	-1.12	0.038893
Gm15800	predicted gene 15800	502.4	0.46	-1.13	0.043195
Lss	lanosterol synthase	154.1	0.44	-1.19	0.046385
Tuba1c	tubulin, α1C	1779.9	0.44	-1.19	0.037291
Timp2	tissue inhibitor of metalloproteinase 2	294.8	0.44	-1.2	0.04697
Tmsb10	thymosin, β10	473.7	0.43	-1.22	0.04194
Fdft1	farnesyl diphosphate farnesyl transferase 1	312.1	0.43	-1.22	0.03784
Hsbp1	heat shock factor-binding protein 1	362.8	0.43	-1.22	0.033025
Arrb1	arrestin, β1	297.2	0.43	-1.22	0.031968
Igdcc4	immunoglobulin superfamily, DCC subclass, member 4	182.2	0.42	-1.24	0.036855
Trak1	trafficking protein, kinesin-binding 1	279	0.41	-1.28	0.047585
Usp13	ubiquitin-specific peptidase 13 (isopeptidase T-3)	85.7	0.41	-1.3	0.041395
Gm6682	tubulin, α1C pseudogene	673.2	0.41	-1.3	0.026988
Ptgfrn	prostaglandin F_2 receptor negative regulator	229.2	0.39	-1.36	0.042508
Asns	asparagine synthetase	119.7	0.39	-1.37	0.019912
Rnf187	ring-finger protein 187	126.5	0.38	-1.41	0.037146
Wbscr17	Williams-Beuren syndrome chromosome region 17 homolog (*human*)	44.7	0.35	-1.5	0.041549
Clip3	CAP-GLY domain-containing linker protein 3	196.3	0.35	-1.51	0.013397
Sulf2	sulfatase 2	187.1	0.35	-1.53	0.027623
Bpgm[f]	2,3-*bis*-phosphoglycerate mutase	378.1	0.35	-1.53	0.026492
Fam210b	family with sequence similarity 210, member B	61.8	0.35	-1.53	0.025588
Pid1	phosphotyrosine interaction domain-containing 1	182.1	0.34	-1.54	0.022869
Ankrd44	ankyrin-repeat domain 44	501.4	0.34	-1.57	0.0437
Xk	Kell blood group precursor (McLeod phenotype) homolog	128.4	0.33	–1.60	0.016258
Endod1	endonuclease domain-containing 1	55.8	0.33	-1.61	0.015329
Scd1	stearoyl-Coenzyme A desaturase 1	82.6	0.33	-1.62	0.015271
Tnc	tenascin C	514.9	0.32	-1.63	0.032698
Grina	glutamate receptor, ionotropic, N-CH_3-D-Asp-assoc protein 1 (Glx binding)	31.9	0.31	-1.68	0.035333
Gpr173	G-protein-coupled receptor 173	25.5	0.3	-1.72	0.041552
Tmem56	transmembrane-protein 56	181.8	0.3	-1.73	0.032215
Epb4.1l3	erythrocyte protein band 4.1-like 3	379.8	0.3	–1.73	0.011557
Prune2	prune homolog 2 (*Drosophila*)	72.8	0.29	-1.76	0.045509
Palmd	palmdelphin	39.3	0.28	-1.81	0.012063
Stim1	stromal-interaction molecule 1	44	0.28	-1.81	0.027134
Lair1	leukocyte-associated Ig-like receptor 1	18.5	0.28	-1.84	0.040326
Amy2a5	amylase 2a5	52	0.28	-1.86	0.022358
Nkain1	Na+/K+ transporting ATPase-interacting 1	50.1	0.27	-1.9	0.039634
Masp1	mannan-binding lectin serine peptidase 1	33.6	0.26	-1.94	0.014467
Ddc	DOPA decarboxylase	52.6	0.26	-1.95	0.019292
Ndrg2	N-myc downstream-regulated gene 2	460.2	0.25	-1.99	0.046684
Vat1	vesicle amine-transport protein 1 homolog (*T. californica*)	40.6	0.25	-2.01	0.00859
Paqr9	progestin and adipoQ receptor family member IX	81.8	0.25	-2.02	0.02769
Galnt16	UDP-*N*-acetyl-α-D-galactosamine:polypeptide *N*-acetyl-galactosamine transferase 16	53.8	0.24	-2.06	0.025983
Mageb16-ps1	melanoma antigen family B, 16, pseudogene 1	50.5	0.24	-2.06	0.038005
Ajap1	adherens junction-associated protein 1	15.7	0.24	-2.07	0.045122
Hspb6	heat-shock protein, α-crystallin-related, B6	16.7	0.24	-2.08	0.046724
Speer8-ps1	spermatogenesis-associated glutamate (E)-rich protein 8, pseudogene 1	13.8	0.22	-2.17	0.040374
Mras	muscle and microspikes RAS	71.6	0.22	-2.21	0.024459
Me1	malic enzyme 1, NADP(+)-dependent, cytosolic	149	0.21	-2.23	0.031703
Slc1a4	solute carrier family 1 (glutamate/neutral amno acid transporter), member 4	81.4	0.21	–2.25	0.024939

Tox	thymocyte selection-associated high-mobility group box	60.1	0.2	-2.3	0.006862
Tal1	T-cell acute lymphocytic leukemia 1	52.2	0.2	–2.30	0.010111
Slc2a3	solute carrier family 2 (facilitated glucose transporters), member 3	169.1	0.2	–2.31	0.030869
Myh8	myosin, heavy polypeptide 8, skeletal muscle, perinatal	15.5	0.19	-2.43	0.031745
Sptb	spectrin β, erythrocytic	62.5	0.18	–2.46	0.01178
Mirg	miRNA-containing gene	23.9	0.18	-2.5	0.025552
Tmcc2	transmembrane and coiled-coil domains 2	26.7	0.17	-2.52	0.019628
Atp7b	ATPase, Cu++ transporting, β polypeptide	8.8	0.17	-2.54	0.046861
6430411K18Rik	RIKEN cDNA 6430411K18 gen	35.1	0.17	-2.56	0.047039
Zcchc16	zinc-finger, CCHC domain-containing 16	8.3	0.16	-2.62	0.042393
Rian	RNA-imprinted and -accumulated in nucleus	1973.2	0.16	-2.62	0.025825
Col19a1	collagen, type XIX, α1	17.1	0.16	-2.63	0.036613
Mab21l2	mab-21-like 2 (*C. elegans*)	36.2	0.16	-2.67	0.013764
Slc22a4	solute carrier family 22 (organic cation transporter), member 4	19.5	0.15	–2.70	0.022468
Aldh1l2	aldehyde dehydrogenase family 1, member L2	239	0.15	-2.74	0.025841
Tmem132c	transmembrane-protein 132C	17.9	0.15	-2.78	0.014959
Rundc3a	RUN domain-containing 3A	8.1	0.14	-2.81	0.042826
Qpct	glutaminyl-peptide cyclotransferase (glutaminyl cyclase)	30.1	0.14	-2.82	0.022509
Snca	synuclein, α	153.6	0.14	-2.86	0.010375
AF357425	snoRNA AF357425	96.9	0.14	-2.88	0.042269
Aldh1a1	aldehyde dehydrogenase family 1, subfamily A1	183.2	0.14	-2.88	0.011707
DQ267100	snoRNA DQ267100	112.1	0.14	-2.88	0.023599
Rpp25	ribonuclease P/MRP 25 subunit	8.2	0.12	-3.01	0.032914
AF357359	snoRNA AF357359	58.1	0.12	-3.04	0.023706
Aff2	AF4/FMR2 family, member 2	185.6	0.12	-3.1	0.012867
Optc	Opticin	7.2	0.11	-3.2	0.02907
Mageb16	melanoma-antigen family B, 16	137.7	0.09	-3.42	0.013263
Grem2	gremlin 2 homolog, cysteine-knot superfamily (*Xenopus laevis*)	46.9	0.09	-3.47	0.017832
Pex5l	peroxisomal biogenesis factor 5-like	13.2	0.08	-3.6	0.022658
A2m	α2-macroglobulin	39.7	0.08	-3.68	0.048256
Mir1906-1	microRNA 1906-1	7.9	0.08	-3.71	0.011924
Mir1906-2	microRNA 1906-2	7.9	0.08	-3.71	0.011924
Ank1	ankyrin 1, erythroid	81.8	0.08	–3.73	0.017692
2810459M11Rik	RIKEN cDNA 2810459M11 gene	10.1	0.07	-3.81	0.036795
Slc30a10	solute carrier family 30, member 10	117.8	0.06	–4.06	0.01406
E130309D14Rik	RIKEN cDNA E130309D14 gene	7.9	0.03	-5.03	0.00286
Otof	Otoferlin	6.5	0.03	-5.23	0.011119
Zfhx4	zinc-finger homeodomain 4	805.6	0.02	-5.66	0.007916
Nrn1	neuritin 1	139.2	0.02	-5.76	0.032328
Itgbl1	integrin, β-like 1	39.9	0.02	-5.99	0.008509
Cpne4	copine IV	26.5	0	-7.81	0.022175
Darc	Duffy blood group, chemokine receptor	17.1	NA	–10.00	0.02699
Slc17a6	solute carrier fam 17 (Na-dependent inorganic phosphate cotransporter), member 6	485.5	NA	–10.00	0.042424
Gpr50	G-protein-coupled receptor 50	9.4	NA	-10	0.049046
Lrrc73	Leucine-rich repeat-containing 73	3.8	NA	-10	0.048763
Cdh7	cadherin 7, type 2	126.4	NA	-10	0.038507
Gpr158	G-protein-coupled receptor 158	75.4	NA	-10	0.049454
Cacng3	calcium channel, voltage-dependent, γ subunit 3	7.3	NA	-10	0.024119
Stmn4	stathmin-like 4	113	NA	-10	0.038829
Snord116	small nucleolar RNA, C/D box 116	83.1	NA	-10	0.036871
Snord116l1	small nucleolar RNA, C/D box 116-like 1	83.1	NA	-10	0.036871
Snord116l2	small nucleolar RNA, C/D box 116-like 2	83.1	NA	-10	0.036871
2610100L16Rik	RIKEN cDNA 2610100L16 gene	139	NA	-10	0.036769
Klhl1	kelch-l like	82.5	NA	-10	0.047351

GD16.5 LUNG

**Gene symbol**	**Official gene name**	**Average expression (RPKM)**	**Fold-Change**	**log_2 Fold-Change**	***P*-value**
Gbp1	guanylate-binding protein 1	5.9	28.81	**+4.85**	0.036787
Wnt7a	wingless-related MMTV integration site 7A	3.1	19.46	4.28	0.027765

Myh3	myosin, heavy polypeptide 3, skeletal muscle, embryonic	70.2	8.32	3.06	0.034623
Cngb1	cyclic nucleotide-gated channel β1	6.8	7.31	2.87	0.006094
Tslp	thymic stromal lymphopoietin	12.4	6.85	2.78	0.001705
Tceal7	transcription elongation factor A (SII)-like 7	13.2	5.55	2.47	0.013361
Atp1b4	ATPase, (Na+)/K+ transporting, β4 polypeptide	23.6	5.53	2.47	0.03129
Krt15	keratin 15	14.7	3.5	1.81	0.008463
Sftpc	surfactant-associated protein C	736.4	3.48	1.8	0.043302
Egfem1	EGF-like and EMI domain-containing 1	109.6	3.37	1.75	0.018341
Ccdc158	coiled-coil domain-containing 158	31.4	2.8	1.49	0.007803
Poln	DNA polymerase N	22.1	2.63	1.4	0.019689
Fas	Fas (TNF receptor superfamily member 6)	18.6	2.56	1.36	0.028566
Ppp1r3c	protein phosphatase 1, regulatory (inhibitor) subunit 3C	737.2	2.53	1.34	0.004868
Frrs1l	ferric-chelate reductase 1-like	62.5	2.45	1.29	0.006612
Rab38	RAB38, member of RAS oncogene family	55.1	2.41	1.27	0.036639
Ager	advanced glycosylation end product-specific receptor	20.5	2.34	1.23	0.038365
Slc26a9	solute carrier family 26, member 9	31.2	2.31	1.21	0.035034
Cebpa	CCAAT/enhancer binding protein (C/EBP), α	19.8	2.29	1.19	0.037194
Dpt	dermatopontin	118.5	2.28	1.19	0.038236
Abcb1a	ATP-binding cassette, sub-family B (MDR/TAP), member 1A	66.7	2.24	1.16	0.018031
Lgr6	Leu-rich repeat-containing G-protein-coupled receptor 6	25	2.22	1.15	0.018926
Myh14	myosin, heavy polypeptide 14	26.1	2.2	1.14	0.018232
Scn3b	sodium channel, voltage-gated, type III, β	76.5	2.2	1.13	0.001647
AF357359	snoRNA AF357359	54	2.16	1.11	0.004584
Pirt	phosphoinositide-interacting regulator of transient-receptor potential channels	49.1	2.01	1.01	0.03256
Lpcat1	lysophosphatidylcholine acyltransferase 1	217.3	2	1	0.012104
4930570G19Rik	RIKEN cDNA 4930570G19 gene	27.3	0.47	–1.09	0.043599
Cyp2s1	**cytochrome P450, family 2, subfamily s, polypeptide 1**	25.9	0.46	1.11	0.026306
Negr1	neuronal growth regulator 1	181.9	0.45	-1.14	0.011947
Zfp641	zinc-finger protein 641	24.2	0.45	-1.15	0.044072
Epb4.2	erythrocyte protein band 4.2	29.6	0.43	-–1.21	0.027399
Tuba4a	tubulin, α 4A	27.4	0.42	-1.24	0.021661
Frrs1	ferric-chelate reductase 1	44.8	0.42	-1.25	0.012293
Rgn	Regucalcin	52.7	0.4	-1.31	0.000861
Capn5	calpain 5	18.9	0.39	-1.35	0.049831
F11	coagulation factor XI	9.8	0.39	-1.35	0.049162
Cfb	complement factor B	37.7	0.39	-1.36	0.006608
Ppargc1b	peroxisome proliferative-activated receptor γ, coactivator 1β	19	0.38	-1.38	0.031338
Slc4a1 [g]	solute carrier family 4 (anion exchanger), member 1	173	0.38	–1.41	0.034826
Cyp3a16	**cytochrome P450, family 3, subfamily a, polypeptide 16**	8.8	0.36	–1.46	0.03581
Got1	glutamate oxaloacetate transaminase 1, soluble	65.9	0.36	-1.47	0.017347
Ermap	erythroblast membrane-associated protein	109.8	0.36	–1.48	0.023993
Rbp4	retinol-binding protein 4, plasma	29.5	0.35	-1.53	0.001504
Gys2	glycogen synthase 2	12.5	0.34	-1.57	0.012228
Acpp	acid phosphatase, prostate	22.7	0.33	-1.59	0.026206
C8b	complement component 8, β polypeptide	18.7	0.33	-1.6	0.002955
Itih2	inter-α trypsin inhibitor, heavy chain 2	138.9	0.33	-1.61	0.000457
Syt9	synaptotagmin IX	11.9	0.32	-1.66	0.049333
Cps1	carbamoyl-phosphate synthetase 1	26.9	0.31	-1.68	0.012572
Prr15	proline-rich 15	11.1	0.3	-1.73	0.041482
Tdrd5	tudor domain-containing 5	21.7	0.3	-1.74	0.03502
Trf	transferrin	244.4	0.29	–1.78	0.001129
F13b	coagulation factor XIII, β subunit	16.8	0.29	-1.78	0.002825
Alb	albumin	2289.8	0.28	-1.81	0.000304
Otc	ornithine transcarbamylase	11.1	0.28	-1.85	0.007028
Tmem56	transmembrane-protein 56	78.6	0.27	-1.88	7.98E-05
Gc	group-specific component	25	0.27	-1.89	0.000189
Slc22a4	solute carrier family 22 (organic cation transporter), member 4	9.7	**0.27**	**–1.91**	**0.036427**
Edaradd	EDAR (ectodysplasin-A receptor)-associated death domain	46	0.27	-1.91	0.021752
Gsta3	glutathione *S*-transferase, a3	28.6	0.27	-1.92	0.002929
Bhmt	betaine-homocysteine methyltransferase	15.3	0.26	-1.92	0.002084
Cyp2c67	**cytochrome P450, family 2, subfamily c, polypeptide 67**	7.4	0.26	–1.93	0.026034
Apob	apolipoprotein B	717.9	0.26	-1.93	0.00013

Apoh	apolipoprotein H	10.6	0.26	-1.96	0.006327
Afp	α-fetoprotein	569.1	0.26	-1.96	5.30E-06
Klkb1	kallikrein B, plasma 1	8.1	0.26	-1.97	0.02064
F5	coagulation factor V	40	0.25	-1.99	0.000185
Slco5a1	solute carrier organic anion transporter family, member 5A1	40.3	0.25	–1.99	0.047989
Aldh8a1	aldehyde dehydrogenase 8 family, member A1	7.3	0.25	-2.01	0.025139
Hemgn	hemogen	133	0.25	–2.02	0.008414
Ahsg	α2-HS-glycoprotein	68.3	0.24	-2.05	6.85E-07
Cyp2c40	**cytochrome P450, family 2, subfamily c, polypeptide 40**	10.1	0.24	–2.08	0.006742
Apoa2	apolipoprotein A-II	25.3	0.24	-2.09	0.001639
Serpina1d	serine (or cysteine) peptidase inhibitor, clade A, member 1D	20.3	0.24	-2.09	0.000281
Pzp	pregnancy-zone protein	121.7	0.23	-2.09	2.53E-06
Akr1d1	aldo-keto reductase family 1, member D1	6.4	0.23	-2.12	0.036532
Apoa1	apolipoprotein A-I	59.8	0.23	-2.15	8.22E-05
Serpina1c	serine (or cysteine) peptidase inhibitor, clade A, member 1C	25.8	0.22	-2.16	2.67E-05
Hsd17b2	hydroxysteroid (17β) dehydrogenase 2	5.5	0.21	-2.22	0.028871
Serpina1e	serine (or cysteine) peptidase inhibitor, clade A, member 1E	18.6	0.21	-2.23	0.000263
Pklr	pyruvate kinase liver and red blood cell	6.5	0.21	–2.24	0.027325
Rab11fip4	RAB11 family-interacting protein 4 (class II)	7.5	0.21	-2.25	0.029383
Fgg	fibrinogen γ chain	125.3	0.21	-2.25	1.10E-08
Serpina1b	serine (or cysteine) preptidase inhibitor, clade A, member 1B	40.9	0.21	-2.25	4.19E-07
Ttr	Transthyretin	61.2	0.21	-2.25	8.56E-08
Fn3k	Fructosamine-3 kinase	14.1	0.21	-2.25	0.020082
Rprm	reprimo, TRP53-dependent G_2 arrest mediator candidate	12.8	0.21	-2.27	0.025739
Timd2	T-cell immunoglobulin- and mucin domain-containing 2	9.1	0.2	-2.29	0.010745
Vtn	Vitronectin	8.3	0.2	-2.33	0.006947
Kng1	kininogen 1	41.5	0.2	-2.35	9.57E-08
Fgb	fibrinogen β-chain	96.2	0.2	-2.36	1.06E-10
C3	complement component 3	60.7	0.19	-2.37	0.000239
Fga	fibrinogen α chain	90.3	0.19	-2.39	3.55E-10
Olfr78	olfactory receptor 78	5.4	0.19	-2.39	0.044948
Slc14a1	solute carrier family 14 (urea transporter), member 1	7.8	0.19	–2.39	0.01236
Plg	plasminogen	15.7	0.18	-2.44	0.000143
Cyp2c68	**cytochrome P450, family 2, subfamily c, polypeptide 68**	14.7	0.18	–2.51	0.000346
Slc30a10	solute carrier family 30, member 10	30.6	0.17	–2.52	0.023084
Serpina6	serine (or cysteine) peptidase inhibitor, clade A, member 6	72.8	0.17	-2.55	4.07E-09
Dsc2	desmocollin 2	70.1	0.16	-2.67	0.034503
Mcoln2	mucolipin 2	7.6	0.16	-2.67	0.018071
Fabp1	fatty-acid binding-protein 1, liver	6.4	0.15	-2.69	0.013204
Aadac	arylacetamide deacetylase (esterase)	33	0.15	-2.75	0.00991
C8a	complement component 8, α polypeptide	6.3	0.14	-2.81	0.00857
Ugt2b34	UDP glycosyltransferase 2 family, polypeptide B34	51.9	0.13	-2.89	0.007348
Dpp10	dipeptidylpeptidase 10	7.2	0.13	-2.95	0.01963
Abcb11	ATP-binding cassette, sub-family B (MDR/TAP), member 11	5	0.09	-3.46	0.008631
Ambp	α1 microglobulin/bikunin	15.3	0.09	-3.5	3.98E-06
Dmrt2	doublesex- and mab-3-related transcription factor 2	3.9	0.09	-3.53	0.039071
Itih3	inter-α trypsin inhibitor, heavy chain 3	25.2	0.08	-3.56	2.61E-08
Uroc1	urocanase domain-containing 1	3.1	0.08	-3.57	0.033389
Skap1	Src family-associated phosphoprotein 1	4.3	0.08	-3.69	0.03618
Spp2	secreted phosphoprotein 2	15.1	0.08	-3.71	0.045364
Amy2b	amylase 2b	2.8	0.06	-3.95	0.0494
Ctse	cathepsin E	23.2	0.06	-4.03	0.004886
Slc5a9	solute carrier family 5 (sodium/glucose cotransporters), member 9	10.3	0.01	–6.21	0.044942
Thy1	thymus cell antigen 1, Θ	13.3	0.01	-6.59	0.007509
Hoxd11	homeobox D11	137.9	NA	-10	0.040832
Itih1	inter-α trypsin inhibitor, heavy chain 1	2.2	NA	-10	0.035079
Acot12	acyl-CoA thioesterase 12	2.5	NA	-10	0.025818
**GD16.5 HEART**					
**Gene symbol**	**Official gene name**	**Average expression (RPKM)**	**Fold-Change**	**$\log_2$ Fold-Change**	***P*-value**

AU018829	expressed sequence AU018829	1.8	NA	**+10**	0.026286
Gm3286	predicted gene 3286	2.5	NA	10	0.008466
Ager	advanced glycosylation end product-specific receptor	4.9	NA	10	3.29E-05
Scgb3a2	secretoglobin, family 3A, member 2	2.6	NA	10	0.041262
Cbr2	carbonyl reductase 2	3.4	NA	10	0.017131
Crabp2	cellular retinoic acid-binding protein II	1.6	NA	10	0.047249
Cyp2f2	**cytochrome P450, family 2, subfamily f, polypeptide 2**	4.8	NA	10	0.021909
Cd74	CD74 antigen (invariant polypeptide of MHC, class II antigen-associated)	16.1	NA	10	0.001655
Inmt	indolethylamine *N*-methyltransferase	3.9	NA	10	0.044646
Scgb1a1	secretoglobin, family 1A, member 1 (uteroglobin)	62.6	NA	10	0.000148
Cd52	CD52 antigen	2.8	NA	10	0.045474
Gm16367	predicted gene 16367	1.6	NA	10	0.047249
Sftpc	surfactant-associated protein C	27.4	46.49	5.54	0.009319
Gbp1	guanylate-binding protein 1	4.5	24.03	4.59	0.036024
Slc34a2	solute carrier family 34 (sodium phosphate), member 2	2.7	17.77	4.15	0.031583
H2-Eb1	histocompatibility 2, class II antigen E β1	7.4	13.67	3.77	0.019073
Psmb8	proteasome (prosome, macropain) subunit, β type 8 (multifunctnl peptidase 7)	6.1	9.82	3.3	0.022277
Mir682	microRNA 682	4.3	8.97	3.17	0.042703
Vill	villin-like	4.3	8.91	3.16	0.009913
Gpr97	G-protein-coupled receptor 97	3.9	5.38	2.43	0.045928
Itga2b	integrin α2b	4.7	4.32	2.11	0.028463
Dbc1	deleted in bladder cancer 1 (*human*)	6.5	3.7	1.89	0.020692
Dab1	disabled 1	8.1	3.61	1.85	0.022708
2700089I24Rik	RIKEN cDNA 2700089I24 gene	7.9	3.39	1.76	0.017813
BC024139	cDNA sequence BC024139	10.3	3.36	1.75	0.011219
Usp43	ubiquitin-specific peptidase 43	7.6	3.29	1.72	0.030229
Lgals3bp	lectin, galactoside-binding, soluble, 3 binding protein	7.4	3.15	1.66	0.026976
AI837181	expressed sequence AI837181	7.1	2.87	1.52	0.049133
Acap1	ArfGAP with coiled-coil, ankyrin repeat and PH domains 1	6.8	2.84	1.51	0.029801
Zfpm1	zinc-finger protein, multitype 1	8.2	2.8	1.49	0.037439
Ephx1	epoxide hydrolase 1, microsomal	14.5	2.73	1.45	0.011254
Shb	Src homology 2 domain-containing transforming protein B	13.2	2.55	1.35	0.014603
Ybx2	Y-box protein 2	17.5	2.49	1.31	0.007175
Sftpb	surfactant-associated protein B	25.1	2.46	1.3	0.042026
Emc10	ER-membrane protein complex subunit 10	17.7	2.43	1.28	0.005151
Zfhx2	zinc-finger homeobox 2	16.1	2.33	1.22	0.015254
Hoxb4	homeobox B4	19.4	2.24	1.17	0.02096
Ust	uronyl-2-sulfotransferase	10.5	2.22	1.15	0.048486
Syp	Synaptophysin	18.5	2.19	1.13	0.019403
Ddx49	DEAD (Asp-Glu-Ala-Asp) box polypeptide 49	14.1	2.18	1.13	0.019958
Bud13	BUD13 homolog (*yeast*)	25.2	2.12	1.08	0.005948
Erdr1	erythroid differentiation regulator 1	111.2	2.05	1.04	0.046802
Gpr65	G-protein-coupled receptor 65	31.2	0.5	–1.01	0.007421
Slc25a37[h]	solute carrier family 25, member 37	2057.6	0.45	–1.14	3.49E-05
Fam46c	family with sequence similarity 46, member C	70.2	0.45	-1.16	0.022372
Cldn11	claudin 11	24.1	0.44	-1.2	0.048754
Hsd3b6	hydroxy-Δ-5-steroid dehydrogenase, 3β- and steroid Δ-isomerase 6	10.7	0.35	-1.49	0.019907
Sowaha	sosondowah-ankyrin repeat-domain family member A	12.6	0.35	-1.53	0.023357
Isg20	interferon-stimulated protein	10.8	0.33	-1.62	0.039091
Mirlet7d	microRNA let7d	4.9	0.29	-1.81	0.028989
4930570G19Rik	RIKEN cDNA 4930570G19 gene	4.3	0.15	-2.71	0.013752
Il1rapl2	interleukin 1 receptor accessory protein-like 2	5	0.15	-2.74	0.003039
Ms4a2	membrane-spanning 4-domains, subfamily A, member 2	3	0.07	-3.77	0.018637

GD16.5 CEREBELLUM

**Gene symbol**	**Official gene name**	**Average expression (RPKM)**	**Fold-Change**	**$\log_2$ Fold-Change**	***P*-value**
Btbd11	BTB (POZ) domain-containing 11	58.1	2.58	**+1.37**	0.04094
Cyp1b1	**cytochrome P450, family 1, subfamily b, polypeptide 1**	52	0.4	–1.31	0.034512

Naa38	*N*-α-acetyltransferase 38, NatC auxiliary subunit	129.4	0.38	-1.4	0.043117
Spta1	spectrin α, erythrocytic 1	28	0.29	-1.78	0.025538
Mrc1	mannose receptor, C-type 1	111.1	0.27	-1.87	0.036005
Cfhr2	complement factor H-related 2	34.9	0.25	-2.01	0.04028
Apob	apolipoprotein B	86.2	0.23	-2.1	0.035616
Cfh	complement-component factor h	284.3	0.23	-2.11	0.018478
Dcn	decorin	49.1	0.19	-2.38	0.001055
Lum	lumican	14.7	0.14	-2.87	0.000786
Vamp5	vesicle-associated membrane protein 5	11.1	0.1	-3.37	0.017568
Tlr7	toll-like receptor 7	5.8	0.09	-3.52	0.015934
Aoah	acyloxyacyl hydrolase	3.3	0.04	-4.67	0.045123
Tspan8	tetraspanin 8	202.2	NA	-10	0.046697
Il21r	interleukin 21 receptor	2.6	NA	-10	0.02836

[a] More than 80 genes in ***bolded red font*** denote hematopoiesis- and hypoxia-related genes, including iron transport**;** these include all platelet and white cell types and functions because all are derived from hematopoietic stem cells. Yolk sac and lung had the most hematopoiesis- and hypoxia-related genes (about two dozen each)**;** liver showed the least, only three genes. Genes in ***bolded blue font*** include those in the solute carrier (*Slc*) superfamily and other transporter and channel families. Genes in ***bolded green font*** are members of the cytochrome P450 (*Cyp*) gene superfamily.

[b] Two *Gbp* genes coding for guanylate-binding proteins were strikingly up-regulated in six *Slc39a8(neo/neo)* tissues**:** *Gbp1* in yolk sac, placenta, liver, lung and heart**;** *Gbp3* in placenta. GBPs play pivotal roles in many critical-life processes including inflammatory and innate immune functions**;** the GBP family comprises seven members and is a subset within the dynamin-related large GTPases that are expressed in response to interferons and other pro-inflammatory cytokines[1]. In addition to GBPs being induced by interferon, and protecting against intracellular pathogens, a growing number of GBPs are not directly related to pathogen protection[2]. Our discovery of dramatically up-regulated *Gbp* genes in five of seven *Slc39a8(neo/neo)* tissues examined — would support this view that GBPs are involved in much more than just defense against inflammation.

[c] Insulin-like growth factor (IGF)-binding proteins were up-regulated in yolk sac. Hypoxia is known to induce IGF-binding proteins and stimulate erythropoietin formation.

[d] *Npr3* (natriuretic peptide receptor-3) is up-regulated, and this finding is relevant to another investigation in this laboratory. In a *Sglt2>Cre>Slc39a8(flox/neo)* conditional knockout in which renal epithelial ZIP8 was ablated, atrial natriuretic peptide (ANP) levels were markedly decreased (Jorge-Nebert L, Soleimani M, and Nebert DW, *unpublished*). Atrial natriuretic peptides (ANPs) are known to dilate blood vessels and stimulate natriuresis and diuresis — rsulting in lowered blood pressure and blood volume**;** in part, ANPs counterbalance actions of the renin-angiotensin-aldosterone and neurohormonal systems, and play a pivotal role in cardiovascular regulation[3]. In liver, *Ace2* (angiogenesis I-converting enzyme) was also up-regulated, contributing perhaps to the potential clinical role of *SLC39A8* and heart disease.

A genome-wide association study (GWAS) of coronary artery disease in almost 104,000 participants (cases vs controls) from eight populations[4] found a significant association ($P \leq 5.0 \times 10^{-8}$) between a putatively functional single-nucleotide variant in the *SLC39A8* gene and serum levels of high-density-lipoprotein cholesterol. In the human and mouse genomes, there are three *ANP* genes and three natriuretic peptide receptor genes (*NPR1*, *NPR2*, *NPR3*). If ZIP8 deficiency results in ANP depletion, it seems plausible that up-regulation of the receptor *Npr3* might follow. We speculate that enhanced renal *Npr3* in the *Slc39a8(neo/neo)* fetus might therefore be relevant to the GWAS — implicating a relationship between the *SLC39A8* variant and coronary heart disease.

[e] *Ace2* (angiotensin I-converting enzyme A2) is up-regulated in kidney**;** ACE2 has been associated with the induction of erythrocyte formation.

[f] *Bpgm* encodes BPGM (2,3-*bis*-phosphoglycerate mutase) catalyzes 2,3-diphosphogylycerate — which regulates the affinity of hemoglobin for oxygen. Several variant mutations gene are known clinically to cause hemolytic anemia and affect erythrocyte metabolism**;** this is this case in the Luebering-Rapaport pathway. BPGM, together with GSH and pyruvate, are affected as the result of adaptation of erythrocytes during anemia. Erythrocytes, due to lack of mitochondria, obtain their energy only through anaerobic glycolysis.

[g] *Slc4a1* encodes a red blood cell solute carrier family 4, member 1, a chloride/bicarbonate anion exchanger. One null mutation in *SLC4A1* causes severe anemia, and other mutations are known to detabilize the red cell membrane [*reviewed in Ref. 5*].
[h] *Slc25a37*, down-regulated in heart, encodes a solute carrier that moves Fe^{2+} into the mitochondrion, and is important for synthesis of mitochondrial heme in immature erythrocytes.

Differentially-expressed cytochrome P450 (Cyp) genes. We found nine cytochrome P450 (*Cyp*) genes (***green font***) — *Cyp1b1* differentially-expressed in yolk sac, kidney and cerebellum, whereas the other eight *Cyp* genes were each found in only one tissue. Except for *Cyp7b1* (oxysterol metabolism) and *Cyp1b1*, the remaining seven *Cyp* genes are members of the *Cyp2* and *Cyp3* families.

In the mouse genome there are 103 *Cyp* genes in 18 families — with the *Cyp2*, *Cyp3* and *Cyp4* families greatly expanded — compared with the human genome that has 57 *CYP* genes in 18 families[6,7]. *Slc39a8(neo/neo) Cyp1b1* was up-regulated in yolk sac and kidney, but down-regulated in cerebellum. The results were also mixed with the other eight *Cyp* genes.

How can these data be explained? CYP monooxygenases (in particular, members of *Cyp1*, *Cyp2*, *Cyp3* and *Cyp4* families) participate in the second-messenger lipid-mediator (LM) cascade**;** LMs play important roles in virtually every critical-life function [*reviewed in Refs. 7,8*]. In support of this, zymosan-induced peritonitis in *Cyp1(–/–)* triple-knockout mice resulted in alteration of eight distinct LM metabolic steps, suggesting that one or more of the three CYP1 enzymes contribute to the local metabolite profile of LMs that regulate inflammation[9].

Hence, defective ZIP8-mediated function evokes changes in hepatic *Cyp3a11*, pulmonary *Cyp2s1*, *Cyp3a16*, *Cyp2c67*, *Cyp2c40*, and *Cyp2c68*, and cardiac *Cyp2f2* expression levels — which, in turn, will modify cell-specific LM metabolites that regulate numerous critical-life processes. On the other hand, deficient ZIP8-mediated stimulation of *Cyp7b1*, coding for oxysterol 7α-hydroxylase[7], is most likely associated with dysregulation of fatty-acid and steroid homeostasis. Except for *Cyp7b1*, the other eight *Cyp* genes are known to be regulated by AHR/ARNT heterodimeric binding to the AHRE casettte**;** involvement of the AHR/CYP1 axis as “early first-responders to various types of stress signals” has recently been reviewed[10].

Differentially-expressed solute-carrier (Slc) genes. Excluding *Slc39a8*, there were 27 other genes in the solute-carrier (*Slc*) transporter superfamily (***blue font***), some found in two or three of the seven tissues, but most of them differentially-expressed in just one tissue**;** 18 various *Slc* genes were up-regulated and 12 down-regulated, in no discernible pattern. Twenty-one of the 52 known *Slc* gene families were represented, and none of the fourteen genes in the *Slc39* family (other than *Slc39a8*) was differentially-expressed.

The *Slc* gene superfamily contains 57 gene families encoding at least 395 protein-coding genes [https://www.genenames.org/]. SLC proteins include passive transporters, symporters and antiporters, located in all cellular and organelle membranes. Transport substrates include**:** amino acids and oligopeptides, glucose and other sugars, innumerable inorganic cations and anions, bile salts, carboxylate and other organic anions, acetyl coenzyme A, biogenic amines, neurotransmitters, vitamins, fatty acids and lipids, nucleosides, ammonium, choline, thyroid hormone, and urea [*reviewed in Ref. 11*].

In our RNA-seq analysis, besides *Slc39a8* down-regulation, we found *Slc30a10* (as the only one of ten *Slc30* family members) up-regulated in yolk sac and down-regulated in kidney and lung (*vide supra*). SLC30 (ZnT) zinc transporters move Zn^{2+} out of the cell whereas ZIP8 moves Zn^{2+} into the cell, providing a balance between intracellular and extracellular Zn^{2+} concentrations[12]. Other than *Slc39a8* and *Slc30a10* — diminished ZIP8 function resulted in 17 other *Slc* genes up-regulated and 11 other *Slc* genes down-regulated in one or another of the tissues — except for cerebellum. To understand these effects of ZIP8 deficiency on regulation of all these other SLC transporters will require further study. Of interest, other differentially-expressed channel genes (*e.g.* Ca^{2+} and Na^{+}) were also seen in substantial numbers (*vide supra).*

TABLE S2. Genes differentially-expressed in more than one of the seven tissues examined

Genes differentially regulated in at least three tissues

		Fold-change, comparing *Slc39a8(neo/neo)* with *Slc39a8(+/+)*						
**Gene symbol[a]**	**Official gene name**	**Yolk sac**	**Placenta**	**Liver**	**Kidney**	**Lung**	**Heart**	**Cerebellum**
Gbp1	guanylate-binding protein 1	+19.23	+5.69	+6.35		+28.81	+24.03	
Cyp1b1	cytochrome P450, family 1, subfamily b, polypeptide 1	+5.37			+2.26			–2.49
Hemgn	hemogen	+3.83	+18.87			–4.05		
Slc30a10	solute carrier family 30, member 10	+3.59			–16.64	–5.74		
Ank1	ankyrin 1, erythroid	+3.53	+7.76		–13.28			
Bhmt	betaine-homocysteine methyltransferase	–2.20		–2.25		–3.78		
Cfh	complement-component factor h	–2.46			+3.02			–4.32

Genes differentially regulated in at least two tissues

		Fold-change, comparing *Slc39a8(neo/neo)* with *Slc39a8(+/+)*						
**Gene symbol**	**Official gene name**	**Yolk sac**	**Placenta**	**Liver**	**Kidney**	**Lung**	**Heart**	**Cerebellum**
Gbp1	guanylate-binding protein 1	+19.23	+5.69	+6.35		+28.81	+24.03	
Cyp1b1	cytochrome P450, family 1, subfamily b, polypeptide 1	+5.37			+2.26			–2.49
Esm1	endothelial cell-specific molecule 1	+4.62			+3.74			
Hemgn	hemogen	+3.83	+18.87			–4.05		
Slc30a10	solute carrier family 30, member 10	+3.59			–16.64	–5.74		
Ank1	ankyrin 1, erythroid	+3.53	+7.76		–13.28			
Capn5	calpain 5	+3.07				–2.55		
Ermap	erythroblast membrane-associated protein	+2.99				–2.79		
Bcl11a	B-cell CLL/lymphoma 11A (zinc-finger protein)	+2.84	+4.28					
Isg20	interferon-stimulated protein	+2.79					–3.07	
Hsd3b6	HO-Δ-5-steroid dehydrogenase, 3β- and steroid-Δ-isomerase 6	+2.75					–2.82	
Sulf2	sulfatase 2	+2.68			–2.88			
Asns	asparagine synthetase	+2.56			–2.58			
Slc2a3	solute carrier family 2 (facilitated glucose transporters), member 3	+2.50			–4.96			
Gpr50	G-protein-coupled receptor 50	+2.49						
Slc1a4	solute carrier family 1 (Glx/neutral amino acid transporter), member 4	+2.12			–4.77			
Lss	lanosterol synthase	+2.09			–2.28			
Trf	transferrin	+2.05				–3.43		
Aadac	arylacetamide deacetylase (esterase)	–2.04				–6.72		
Bhmt	betaine-homocysteine methyltransferase	–2.20		–2.25		–3.78		
Tlr7	toll-like receptor 7	–2.29						–11.46
Cfh	complement-component factor h	–2.46			+3.02			–4.32
Slc39a8	solute carrier family 39 (metal ion transporter), member 8	–4.06	–3.27					
Hbb-b1	hemoglobin, beta adult major chain	–4.69	–2.53					
Hbb-b2	hemoglobin, beta adult minor chain	–4.69	–2.53					
4930570G19Rik	RIKEN cDNA 4930570G19 gene					–2.12	–6.53	
AF357359	snoRNA AF357359				–8.22	+2.16		
Ager	advanced glycosylation end-product-specific receptor					+2.34		
Apob	apolipoprotein B					–3.80		–4.28
Cfhr2	complement-factor H-related 2				+3.58			–4.04

Cldn11	claudin 11				**+3.21**		**–2.29**	
Cth	cystathionase (cystathionine γ-lyase)			**-2.32**	**+2.56**			
Grina	glutamate receptor, ionotropic, N-methyl-*D*-aspartate-associated protein 1 (Glx- binding)			**+2.62**	**–3.21**			
Hoxd11	homeobox D11				**+2.25**			
Mir1906-1	microRNA 1906-1			**+5.43**	**–13.05**			
Mir1906-2	microRNA 1906-2			**+5.43**	**–13.05**			
Sftpc	surfactant-associated protein C					**+3.48**	**+46.49**	
Slc22a4	solute carrier family 22 (organic cation transporter), member 4				**–6.49**	**–3.77**		
Tal1	T-cell acute lymphocytic leukemia 1		**+3.15**		**–4.93**			
Tmem56	transmembrane-protein 56				**–3.31**	**–3.68**		

[a] Genes are ranked by the degree of differential change in yolk sac. Those with no change seen in yolk sac — are then listed alphabetically below. In the seven *columns* at *right*, up-regulation is denoted in ***red***, down-regulation in ***blue***.

TABLE S3. Significant differentially-expressed genes — using a FDR-adjusted meta-*P*-value <0.1 — that were identified across all tissues by meta-analysis

**Gene symbol**[a]	**Official gene name**	***P*-values for Fisher test FDR down-regulated genes**[b,c]	***P*-values for Fisher test FDR up-regulated genes**[b,c]
Slc39a8	solute carrier family 39 (metal ion transporter), member 8	0.00035	1.00000
Bhmt	betaine-homocysteine methyltransferase	0.00713	1.00000
Hbb-b1	hemoglobin, beta adult major chain	0.00713	1.00000
Hbb-b2	hemoglobin, beta adult minor chain	0.00713	1.00000
Psmc3	proteasome (prosome, macropain) 26S subunit, ATPase 3	0.01182	1.00000
Hbb-bs	hemoglobin subunit beta-1-like	0.01235	1.00000
Apob	apolipoprotein B	0.01235	1.00000
Bpgm[d]	2,3-*bis*-phosphoglycerate mutase	0.02678	1.00000
Cisd2	CDGSH iron sulfur domain 2	0.03351	1.00000
Slc25a37	solute carrier family 25, member 37	0.03451	1.00000
Fgb	fibrinogen β-chain	0.03540	1.00000
Hba-a2	hemoglobin alpha, adult chain 2	0.03540	1.00000
Ahsg	α2-HS-glycoprotein	0.03540	1.00000
Fabp2	fatty-acid-binding protein 2, intestinal	0.03540	1.00000
Hba-a1	hemoglobin alpha, adult chain 1	0.03540	1.00000
Ovca2	candidate tumor suppressor in ovarian cancer 2	0.03540	1.00000
Snca	synuclein, α	0.03930	0.99406
Fgg	fibrinogen γ-chain	0.03930	1.00000
A530054K11Rik	RIKEN cDNA A530054K11 gene	0.03997	1.00000
Cth	cystathionase (cystathionine γ-lyase)	0.04105	0.98646
Hbb-y	hemoglobin Y, beta-like embryonic chain	0.04105	1.00000
Lum	Lumican	0.04105	1.00000
Gsg1l	GSG1-like	0.04105	1.00000
Usmg5	up-regulated during skeletal muscle growth 5	0.05303	1.00000
Slc4a1	solute carrier family 4 (anion exchangers), member 1	0.05759	0.97794
Dgkb	diacylglycerol kinase, β	0.06022	0.98156
Dnajc6	DnaJ (Hsp40) homolog, subfamily C, member 6	0.06022	0.99234
Gm14305	predicted gene 14305	0.06022	1.00000
Zfp758	zinc-finger protein 758	0.06866	1.00000
Gm14308	predicted gene 14308	0.07221	1.00000
Cps1	carbamoyl-phosphate synthetase 1	0.07221	1.00000
Fam166a	family with sequence similarity 166, member A	0.07221	1.00000
6030408B16Rik	RIKEN cDNA 6030408B16 gene	0.07221	1.00000
Actn2	actinin α2	0.07403	1.00000
Slc8a1	solute carrier family 8 (sodium/calcium exchangers), member 1	0.07403	1.00000
Neto2	neuropilin (NRP) & tolloid (TLL)-like 2	0.07403	1.00000
Gpr65	G-protein-coupled receptor 65	0.08457	1.00000
Gucy1a2	guanylate cyclase 1, soluble, α2	0.08457	1.00000
Cdr2[e]	cerebellar degeneration-related 2	0.08709	0.99898
Gm14432	predicted gene 14432	0.08709	1.00000
Xlr	X-linked lymphocyte-regulated complex	0.09410	1.00000
Tomm5	translocase of outer mitochondrial membrane 5 homolog (*Saccharomyces*)	0.09410	1.00000
Paqr9	progestin and adipoQ receptor fam member IX	0.09568	0.97794
Dcn	decorin	0.09568	1.00000
Otc	ornithine transcarbamylase	0.09568	1.00000
Gbp1	guanylate-binding protein 1	1.00000	**<0.00001**
Esm1	endothelial cell-specific molecule 1	1.00000	**0.00725**

[a] Genes in ***bolded blue font*** denote members of the solute carrier (*Slc*) superfamily. Genes in ***bolded red font*** denote hematopoiesis-related genes and genes in other blood cell types and functions.

[b] The last two columns list false discovery rate (FDR)-adjusted *P*-values for down- and up-regulation, respectively. Using this method of analysis, there are 45 genes having an FDR of <0.1 for ***down***-regulation, with *Slc39a8* as the most highly significant *P*-value. Only the last two rows (*Gbp1* and *Esm1* genes) show an FDR of <0.1 for ***up***-regulation.

[c] This meta-analysis combines *P*-values from all tissues into a single *P*-value. In **Step 1**, the genes are ranked from low to high — based on their assigned differential-expression *P*-values in each tissue separately. For example, the rank order of *Slc39a8* in each tissue was**:**

Yolk sac	Placenta	Liver	Kidney	Lung	Heart	Cerebellum
12th	1st	121st	4404th	4783rd	482nd	323rd

In **Step 2**, these rank orders are then converted to empirical *P*-values by dividing the rank by the total number of genes (17,163 in our case), which gives us the *P*-value in each tissue**:**

Yolk sac	Placenta	Liver	Kidney	Lung	Heart	Cerebellum
6.99e-04	5.83e-05	7.05-03	2.57e-01	2.79e-01	2.81e-02	1.88e-02

In **Step 3**, "Fisher's combined probability test" then combines these empirical *P*-values into a single *P*-value. For *Slc39a8*, the combined *P*-value is 2.03e–08. After the FDR adjustment, the combined *P*-value is 0.00035. This *P*-value specifies how likely it is to see, by chance alone, a gene with such a low rank order in seven tissues. Because the genes were ranked from "low" to "high" in **Step 1**, this is a test for "consistent down-regulation" in all seven tissues.

The rank order of genes from "high" to "low" was then calculated by repeating **Steps 1 to 3** in order to create the *P*-values for "consistent up-regulation" in all tissues**;** only the last two genes (*Gbp1*, *Esm1*) in the table are of this category.

This meta-analysis considers all tissues together, and not for any individual tissue. This technique of meta-analysis is widely used in genome-wide association studies and is less commonly used to combine rank-based empirical *P*-values**;** in this case, however, we have used it in combination with ToppGene.

----

[d] It is worth noting that *Bpgm* encodes 2,3-*bis*-phosphoglycerate mutase (BPGM). Clinically, BPGM enzyme deficiency is known to increase the affinity of hemoglobin for oxygen[13].

[e] Intriguingly, the cerebellar degeneration-related-2 gene (*Cdr2*) was down-regulated**;** this finding might be clinically relevant to studies demonstrating cerebellar atrophy and mental retardation in children having a mutant *SLC39A8* allele putatively causing ZIP8 function to be markedly impaired[14,15].

TABLE S4. Known TAL1-downstream target genes found in the present study — comparing *Slc39a8(neo/neo)* with *Slc39a8(+/+)* wild-type.

P-value of <0.05 and absolute fold-change >2 were used as the cut-off values.

**GD13.5 YOLK SAC** ***P* = 1.178e–5**[a]						
**Gene symbol**[b]	**Official gene name**	**Fold-Change**	**$\log_2$ Fold-Change**	***P*val**	**P_{adj}**	**TAL1 effect on transcription**
Egln3	EGL nine homolog 3 (*C. elegans*)	4.218064	+2.076581	4.61E-09	1.51E-05	Activation
Hemgn	hemogen	3.825722	1.935732	5.02E-07	0.001095	Activation
Ank1	ankyrin 1, erythroid	3.527797	1.818768	0.0124	1.00000	Activation
Ermap	erythroblast membrane-associated protein	2.990613	1.580441	0.006552	1.00000	Undetectable
Rhag	Rhesus blood group-associated A glycoprotein	2.515802	1.331018	0.000338	0.331496	Undetectable
Slc2a3	solute carrier family 2 (facilitated glucose transporter), member 3	2.499126	1.321424	0.005112	1.00000	Repression
Hbb-b2	hemoglobin, beta adult minor chain	0.213027	–2.23089	0.01473	1.00000	Repression
**GD13.5 PLACENTA** ***P* = 4.982e–6**						
**Gene symbol**	**Official gene name**	**Fold-Change**	**$\log_2$ Fold-Change**	***P*val**	**P_{adj}**	**TAL1 effect on transcription**
Hemgn	Hemogen	18.87205	+4.238179	2.15E-06	0.045538	Activation
Ank1	ankyrin 1, erythroid	7.760406	2.956132	0.00739	1.00000	Activation
Tal1[c]	T cell acute lymphocytic leukemia 1	3.145147	1.653127	0.038062	1.00000	Undetectable
Ikzf1	IKAROS family zinc-finger 1	2.244527	1.166411	0.033308	1.00000	Undetectable
Hbb-b2	hemoglobin, beta adult minor chain	0.395638	–1.33775	0.028901	1.00000	Repression
Cbfa2t3	core-binding factor, runt domain, α-subunit 2, translocated to, 3 (*human*)	0.042822	-4.54552	0.006988	1.00000	Undetectable
**GD16.5 LIVER** ***P* = 0.2233**						
**Gene symbol**	**Official gene name**	**Fold-Change**	**$\log_2$ Fold-Change**	***P*val**	**P_{adj}**	**TAL1 effect on transcription**
Epor	erythropoietin receptor	3.657651	+1.870917	0.035935	1.00000	Activation
**GD16.5 KIDNEY** ***P* = 0.06134**						
**Gene symbol**	**Official gene name**	**Fold-Change**	**$\log_2$ Fold-Change**	***P*val**	**P_{adj}**	**TAL1 effect on transcription**
Tal1[c]	T-cell acute lymphocytic leukemia 1	0.202956	–2.30076	0.010111	1.00000	Undetectable
Slc2a3	solute carrier family 2 (facilitated glucose transporter), member 3	0.20156	-2.31072	0.030869	1.00000	Repression
Ank1	ankyrin 1, erythroid	0.075312	–3.73098	0.017692	1.00000	Activation
**GD16.5 LUNG** ***P* = 0.01895**						

Gene symbol	Official gene name	Fold-Change	$\log_2$ Fold-Change	*P*val	P_{adj}	TAL1 effect on transcription
Slc4a1	solute carrier family 4 (anion exchanger), membr 1	0.375952	–1.41138	0.034826	1.00000	Activation
Ermap	erythroblast membrane-associated protein	0.358182	-1.48124	0.023993	1.00000	Undetectable
Hemgn	Hemogen	0.246722	-2.01904	0.008414	1.00000	Activation
**GD16.5 HEART *P* = 0.2196**						
**Gene symbol**	**Official gene name**	**Fold-Change**	**$\log_2$ Fold-Change**	***P*val**	**P_{adj}**	**TAL1 effect on transcription**
Zfpm1	zinc-finger protein, multitype 1	2.801525	+1.486213	0.037439	1.00000	Undetectable
**GD16.5 CEREBELLUM *P* = 0.06771**						
**Gene symbol**	**Official gene name**	**Fold-Change**	**$\log_2$ Fold-Change**	***P*val**	**P_{adj}**	**TAL1 effect on transcription**
Spta1	spectrin α, erythrocytic 1	0.291331	–1.77927	0.025538	1.00000	Undetectable

[a] This file contains the Fisher's exact test *P*-values in each tissue, testing for enrichment of TAL1-downstream targets in differentially-expressed genes.

[b] Genes in ***bolded red font*** denote hematopoiesis-related genes and genes in all other blood cell types and functions. Genes in ***bolded blue font*** denote members of the solute carrier (*Slc*) superfamily.

[c] One might ask "How can a gene (*e.g. Tal1*) regulate itself?" Actually, this is not that uncommon in genetic pathways and networks. The gene product (*e.g.* TAL1) participates as a TF that binds upstream in a regulatory module of the gene (*Tal1*) from which it is derived. This phenomenon represents a negative feedback regulatory loop. The effects of TAL1 on downstream targets have been reported[16].

TABLE S5. Known GATA1-downstream target genes found in the present study — comparing *Slc39a8(neo/neo)* with *Slc39a8(+/+)* wild-type. *P*-value of <0.05 and absolute fold-change >2 were used as the cut-off values.

**GD13.5 YOLK SAC** ***P* = 0.001369**[a]					
**Gene symbol**[b]	**Official gene name**	**Fold-Change**	**$\log_2$ Fold-Change**	***P*val**	***P*adj**
Hdac11	histone deacetylase 11	NA	+10.00	0.02759	1.0000
Reln	reelin	4.44	2.15	7.02E-05	0.092
Hemgn	hemogen	3.83	+1.94	5.02E-07	0.001
Slc30a10	solute carrier family 30, member 10	3.59	+1.84	2.38E-05	0.038
Capn5	calpain 5	3.07	1.62	0.03789	1.0000
Ermap	erythroblast membrane-associated protein	2.99	+1.58	0.00655	1.0000
Mthfd2	methylenetetrahydrofolate dehydrogenase (NAD^+-dependent), methenyltetrahydrofolate cyclohydrolase	2.97	1.57	0.00112	0.635
Asns	asparagine synthetase	2.56	1.36	0.00013	0.147
Rhag	Rhesus blood group-associated A glycoprotein	2.52	+1.33	0.00034	0.331
Aldoa	aldolase A, fructose-bisphosphate	2.35	1.24	0.03179	1.0000
Hbb-y	hemoglobin Y, beta-like embryonic chain	0.31	–1.71	2.09E-06	0.004
BC049635	cDNA sequence BC049635	0.29	-1.78	0.03589	1.0000
Slc39a8	solute carrier family 39 (metal ion transporter), member 8	0.25	–2.02	0.00353	1.0000
Hbb-b1	hemoglobin, beta adult major chain	0.21	–2.23	0.01473	1.0000
**GD13.5 PLACENTA** ***P* = 0.02229**					
**Gene symbol**	**Official gene name**	**Fold-Change**	**$\log_2$ Fold-Change**	***P*val**	***P*adj**
Slc43a1	solute carrier family 43, member 1	21.80	+4.45	0.02989	1.0000
Hemgn	Hemogen	18.87	4.24	2.15E-06	0.046
Tal1	T-cell acute lymphocytic leukemia 1	3.15	1.65	0.03806	1.0000
Mns1	meiosis-specific nuclear structural protein 1	2.33	1.22	0.03008	1.0000
Hbb-b1	hemoglobin, beta adult major chain	0.40	–1.34	0.0289	1.0000
Tubb3	tubulin, beta 3 class III	0.38	-1.40	0.02132	1.0000
Slc39a8	solute carrier family 39 (metal ion transporter), member 8	0.31	–1.71	1.94E-05	0.137
Cbfa2t3	core-binding factor, runt domain, $\alpha 2$ subunit, translocated to, 3 (*human*)	0.04	-4.55	0.00699	1.0000
**GD16.5 LIVER** ***P* = 0.6762**					
**Gene symbol**	**Official gene name**	**Fold-Change**	**$\log_2$ Fold-Change**	***P*val**	***P*adj**
Epor	erythropoietin receptor	3.66	+1.87	0.03593	1.0000
Grina	glutamate receptor, ionotropic, N-methyl D-aspartate-associated protein 1 (glutamate binding)	2.62	1.39	0.0421	1.0000

**GD16.5 KIDNEY** ***P* = 0.5224**					
**Gene symbol**	**Official gene name**	**Fold-Change**	**$\log_2$ Fold-Change**	***P*val**	***P*adj**
Asns	asparagine synthetase	0.39	–1.37	0.01991	1.0000
Grina	glutamate receptor, ionotropic, *N*-methyl D-aspartate-associated protein 1 (Glx-binding)	0.31	-1.68	0.03533	1.0000
Tmem56	transmembrane protein 56	0.30	-1.73	0.03222	1.0000
Paqr9	progestin and adipoQ receptor family member IX	0.25	-2.02	0.02769	1.0000
Tal1	T-cell acute lymphocytic leukemia 1	0.20	-2.30	0.01011	1.0000
Rpp25	ribonuclease P/MRP 25 subunit	0.12	-3.01	0.03291	1.0000
Slc30a10	solute carrier family 30, member 10	0.06	–4.06	0.01406	1.0000
**GD16.5 LUNG** ***P* = 0.003366**					
**Gene symbol**	**Official gene name**	**Fold-Change**	**$\log_2$ Fold-Change**	***P*val**	***P*adj**
Lpcat1	lysophosphatidylcholine acyltransferase 1	2.00	+1.00	0.0121	1.0000
Capn5	calpain 5	0.39	–1.35	0.04983	1.0000
Slc4a1	solute carrier family 4 (anion exchanger), member 1	0.38	–1.41	0.03483	1.0000
Ermap	erythroblast membrane-associated protein	0.36	–1.48	0.02399	1.0000
Tmem56	transmembrane protein 56	0.27	-1.88	7.98E-05	0.109
Hemgn	Hemogen	0.25	–2.02	0.00841	1.0000
Pklr	pyruvate kinase liver and red blood cell	0.21	-2.24	0.02733	1.0000
Slc14a1	solute carrier family 14 (urea transporter), member 1	0.19	–2.39	0.01236	1.0000
Slc30a10	solute carrier family 30, member 10	0.17	–2.52	0.02308	1.0000
Ctse	cathepsin E	0.06	-4.03	0.00489	1.0000
**GD16.5 HEART** ***P* = 0.6724**					
**Gene symbol**	**Official gene name**	**Fold-Change**	**$\log_2$ Fold-Change**	***P*val**	***P*adj**
Zfpm1	zinc-finger protein, multitype 1	2.80	+1.49	0.03744	1.0000
Fam46c	family with sequence similarity 46, member C	0.45	–1.16	0.02237	1.0000
**GD16.5 CEREBELLUM** *P* = 0.3652					
**Gene symbol**	**Official gene name**	**Fold-Change**	**$\log_2$ Fold-Change**	***P*val**	***P*adj**
Vamp5	vesicle-associated membrane protein 5	0.10	–3.37	0.01757	1.0000

[a] This file contains the Fisher's exact test *P*-values in each tissue, testing for enrichment of GATA1-downstream targets in differentially-expressed genes.

[b] Genes in ***bolded red font*** denote all hematopoiesis-related genes and genes in other blood cell types and functions. Genes in ***bolded blue font*** denote members of the solute carrier (*Slc*) superfamily.

TABLE S6. Known HIF-downstream targets genes found in the present study — comparing *Slc39a8(neo/neo)* with *Slc39a8(+/+)* wild-type. *P*-value of <0.05 and absolute fold-change >2 were used as the cut-off values.

**GD13.5 YOLK SAC** ***P* = 4.206e–15**[a]					
**Gene symbol**[b]	**Official gene name**	**Fold-Change**	**$\log_2$ Fold-Change**	***P*val**	***P*adj**
Igfbp1	insulin-like growth factor binding protein 1	7.48	+2.90	2.08E-14	4.08E-10
Egln3	EGL nine homolog 3 (*C. elegans*)	4.22	2.08	4.61E-09	1.51E-05
Krt19	keratin 19	2.87	1.52	0.000943	0.600143
Igfbp3	insulin-like growth factor binding protein 3	2.67	1.41	0.00298	1.00000
Slc2a1	solute carrier family 2 (facilitated glucose transporter), member 1	2.55	+1.35	0.000473	0.403399
Bhlhe41	basic helix-loop-helix family, member e41	2.37	1.25	0.010568	1.00000
Aldoa	aldolase A, fructose-bisphosphate	2.35	1.24	0.031794	1.00000
Hk1	hexokinase 1	2.25	1.17	0.024595	1.00000
Tgfb3	transforming growth factor, β3	2.22	1.15	0.008699	1.00000
Bhlhe40	basic helix-loop-helix family, member e40	2.21	1.14	0.006853	1.00000
Gm5506	predicted gene 5506	2.17	1.12	0.024416	1.00000
Pdgfa	platelet-derived growth factor, α	2.07	1.05	0.007183	1.00000
Pkm	pyruvate kinase, muscle	2.06	1.05	0.003951	1.00000
Cited2	Cbp/p300-interacting transactivator, with Glu/Asp-rich COOH-terminal domain, 2	2.06	1.04	0.000622	0.488022
Trf	transferrin	2.05	1.04	0.013549	1.00000
**GD13.5 PLACENTA** ***P* = 1.0000**					
**Gene symbol**	**Official gene name**	**Fold-Change**	**$\log_2$ Fold-Change**	***P*val**	***P*adj**
	NO SIGNIFICANT TARGET GENES FOUND				
**GD16.5 LIVER** ***P* = 1.0000**					
**Gene symbol**	**Official gene name**	**Fold-Change**	**$\log_2$ Fold-Change**	***P*val**	***P*adj**
	NO SIGNIFICANT TARGET GENES FOUND				
**GD16.5 KIDNEY** ***P* = 0.62707**					
**Gene symbol**	**Official gene name**	**Fold-Change**	**$\log_2$ Fold-Change**	***P*val**	***P*adj**
Met	met proto-oncogene	2.30	+1.20	0.022918	1
**GD16.5 LUNG** ***P* = 0.02360**					
**Gene symbol**	**Official gene name**	**Fold-Change**	**$\log_2$ Fold-Change**	***P*val**	***P*adj**

Abcb1a	ATP-binding cassette, sub-family B (MDR/TAP), member 1A	2.24	+1.16	0.018031	1.00000
Cyp2s1	**cytochrome P450, family 2, subfamily s, polypeptide 1**	0.46	–1.11	0.026306	1.00000
Trf	transferrin	0.29	–1.78	0.001129	0.831377
**GD16.5 HEART *P* = 1.0000**					
**Gene symbol**	**Official gene name**	**Fold-Change**	**$\log_2$ Fold-Change**	***P*val**	***P*adj**
	NO SIGNIFICANT TARGET GENES FOUND				
**GD16.5 CEREBELLUM *P* = 1.0000**					
**Gene symbol**	**Official gene name**	**Fold-Change**	**$\log_2$ Fold-Change**	***P*val**	***P*adj**
	NO SIGNIFICANT TARGET GENES FOUND				

[a]This file also contains the Fisher's exact test *P*-values in each tissue, testing for enrichment of HIF-downstream targets in differentially-expressed genes. [b]Genes in ***bolded red font*** denote hematopoiesis-related genes and genes in all derived blood cell types and functions. Genes in ***bolded blue font*** denote members of the solute carrier (*Slc*) superfamily. Gene in ***bolded green font*** denotes a member of the cytochrome P450 (*Cyp*) gene family.

TABLE S7. Significant "top five" categories that surfaced from Ingenuity Pathway Analysis (IPA)

**Top Canonical Pathways**
No significant pathways found

Top Upstream Regulators

Upstream regulator	Molecule type	Predicted activation state	Activation *z*-score[a]	*P*-value of overlap[b]	Target molecules in dataset (alphabetical order)
**HIF1A**	Transcription regulator	Activated	3.699	5.27e–13	ALDOA, APLN, ASNS, BHLHE40, CITED2, EGLN3, ENO1, FSCN1, HIF3A, HMGCL, IGFBP3, KRT19, MIF, PDGFA, PKM, SLC16A3, SLC2A1, SLC2A3, STC2, TGFB3
**Deferoxamine**	Chemical; drug	Activated	2.907	2.59e–10	ALDOA, APLN, BHLHE40, CITED2, EGLN3, ENO1, IGFBP3, ISG20, PKM, SLC2A1, SLC2A3, SPRY4, SULF2
**LINC01139**	Long intergenic non-protein-coding RNA	Activated	2.236	1.91e–09	ALDOA, BHLHE40, IGFBP3, PKM, SLC16A3
**CD38**	Enzyme	Activated	2.47	3.09e–09	ALDOA, ASNS, CITED2, EGLN3, LGALS3, MYADM, PKM, SDC1, SLC16A3, SLC2A1, TUBB2B
**β-estradiol**	Chemical – endogenous mammalian	- - -	1.761	2.32e–08	ALDOA, ANO2, BHLHE40, CCDC170, CITED2, CYP1B1, ENO1, GCH1, GHRH, GPRC5A, HDAC11, HLA-A, HUNK, IGFBP1, IGFBP3, KRT19, LGALS3, MAGEA3 (includes others), MAPT, MGARP, PDGFA, PITPNM2, PKM, PPP1R9B, SDC1, SLC2A1, SLC2A3, STC1, STC2, STK32B, TF, TGFB3

Top Diseases and Biological Functions

Categories	Diseases or functions annotation	FDR adjusted *P*-value	Predicted activation state	Activation *z*-score	Molecules (alphabetical order)	Number of molecules
**Molecular transport**	Transport of molecule	3.05e–05	Increased	3.156	ADRA2B, ANK1, ANO2, AP2A1, APLN, APOC3, ATP5D, BHLHE40, BMP6, CFH, CITED2, CLEC5A, DIO2, GABRA1, GHRH, GRM7, HK1, HLA-A, KCNA4, LSS, MAP1B, MAPT, MIF, MT2, PKM, RELN, RHAG, SLC16A3, SLC1A4, SLC2A1, SLC2A3, SLC38A1, SNAP91, STC1, TF, TGFB3, THOC6, VLDLR	38
**Organismal development**	Morphology of head	3.19e–04	- - -	- - -	ADH7, ADRA2B, CFH, CYP1B1, EGLN3, FSCN1, GHRH, IGFBP1, MAP1B, MAPT, MIF, MT2, PDGFA, PPP1R9B, RELN, SEMA7A, SLC1A4, SLC2A1, SP6, SPRY4, STAMBP, STC1, SULF2, TGFB3, THOC6, TLR7, VLDLR	27
**Carbohydrate metabolism**	Glycolysis of cells	3.19e–04	- - -	1.407	ALDOA, CITED2, ENO1, ESRRB, HK1, MIF, PKM, SLC16A3, SLC2A1	9

**Nervous system – development and function**	Morphology of nervous system	9.78e–04	- - -	- - -	AADAT, ADH7, CFH, CITED2, COL25A1, EGLN3, FSCN1, GABRA1, IGFBP1, MAP1B, MAPT, MIF, MT2, PPP1R9B, RELN, SEMA7A, SLC1A4, SLC2A1, SNAP91, STAMBP, STC1, SULF2, TGFB3, TLR7, VLDLR	25
**Cardiovascular disease; Cell death and survival; Connective tissue disorders; Hematological disease; Hereditary disorder; Organismal injury and abnormalities**	Familial hemolytic anemia	1.29e–03	- - -	- - -	ALDOA, ANK1, CFH, HK1, RHAG	5

[a] The "activation z-score" is used to infer likely activation-states of upstream regulators — based on comparison with a model that assigns random regulation directions.

[b] The *P*-value of overlap calls likely upstream-regulators, based on significant overlap between dataset genes, and known targets that are controlled by a transcription regulator.

Figure S1. Heat map showing $\log_2$ fold-changes of each gene in each tissue. With FDR <0.1 in all tissues, 29 genes were identified. Lung and kidney showed remarkably similar expression changes in these genes (correlation coefficient R^2 = 0.93).

References

1. Praefcke,G.J.K. Regulation of innate immune functions by guanylate-binding proteins. *Int J Med Microbiol.* pii: S1438-4221(17)30425-3, doi: 10.1016/j.ijmm.2017.10.013. [Epub ahead of print] (2018).

2. Vestal,D.J. & Jeyaratnam,J.A. The guanylate-binding proteins**:** emerging insights into the biochemical properties and functions of this family of large interferon-induced guanosine triphosphatase. *J Interferon Cytokine Res.* **31**, 89-97 (2011).

3. Nagai-Okatani,C., Kangawa,K., & Minamino,N. Three molecular forms of atrial natriuretic peptides**:** quantitative analysis and biological characterization. *J Pept. Sci.* **23**, 486-495 (2017).

4. Waterworth,D.M. *et al.* Genetic variants influencing circulating lipid levels and risk of coronary artery disease. *Arterioscler. Thromb. Vasc. Biol.* **30**, 2264-2276 (2010).

5. Reithmeier,R.A. *et al.* Band 3, the human red cell chloride/bicarbonate anion exchanger (AE1, SLC4A1), in a structural context. *Biochim. Biophys. Acta* **1858**, 1507-1532 (2016).

6. Nelson,D.R. *et al.* Comparison of cytochrome P450 (*CYP*) genes from the mouse and human genomes, including nomenclature recommendations for genes, pseudogenes and alternative-splice variants. *Pharmacogenetics* **14**, 1-18 (2004).

7. Nebert,D.W., Wikvall,K., & Miller,W.L. Human cytochromes P450 in health and disease. *Philos. Trans. R. Soc. Lond B Biol Sci.* **368**, 20120431 (2013).

8. Nebert,D.W. & Karp,C.L. Endogenous functions of aryl hydrocarbon receptor (AHR)**:** intersection of cytochrome P450 1 (CYP1)-metabolized eicosanoids and AHR biology. *J Biol Chem* **283**, 36061-36065 (2008).

9. Divanovic,S. *et al.* Contributions of the three CYP1 monooxygenases to pro-inflammatory and inflammation-resolution lipid-mediator pathways. *J Immunol.* **191**, 3347-3357 (2013).

10. Nebert,D.W. Aryl hydrocarbon receptor (AHR): "pioneer member" of the basic-helix/loop/helix per-Arnt-sim (bHLH/PAS) family of "sensors" of foreign and endogenous signals. *Prog. Lipid Res.* **67**, 38-57 (2017).

11. He,L., Vasiliou,K., & Nebert,D.W. Analysis and update of the human solute carrier (SLC) gene superfamily. *Hum Genomics* **3**, 195-206 (2009).

12. Huang,L. & Tepaamorndech,S. The SLC30 family of zinc transporters**:** a review of current understanding of their biological and pathophysiological roles. *Mol. Aspects Med* **34**, 548-560 (2013).

13. Hong,W.J. & Gotlib,J. Hereditary erythrocytosis, thrombocytosis and neutrophilia. *Best. Pract. Res. Clin. Haematol.* **27**, 95-106 (2014).

14. Boycott,K.M. *et al.* Autosomal-recessive intellectual disability with cerebellar atrophy syndrome caused by mutation of the manganese- and zinc-transporter gene *SLC39A8*. *Am J Hum Genet* **97**, 886-893 (2015).

15. Park,J.H. *et al.* SLC39A8 deficiency**:** a disorder of manganese transport and glycosylation. *Am J Hum Genet* **97**, 894-903 (2015).

16. Kassouf,M.T. *et al.* Genome-wide identification of TAL1's functional targets**:** insights into its mechanisms of action in primary erythroid cells. *Genome Res.* **20**, 1064-1083 (2010).

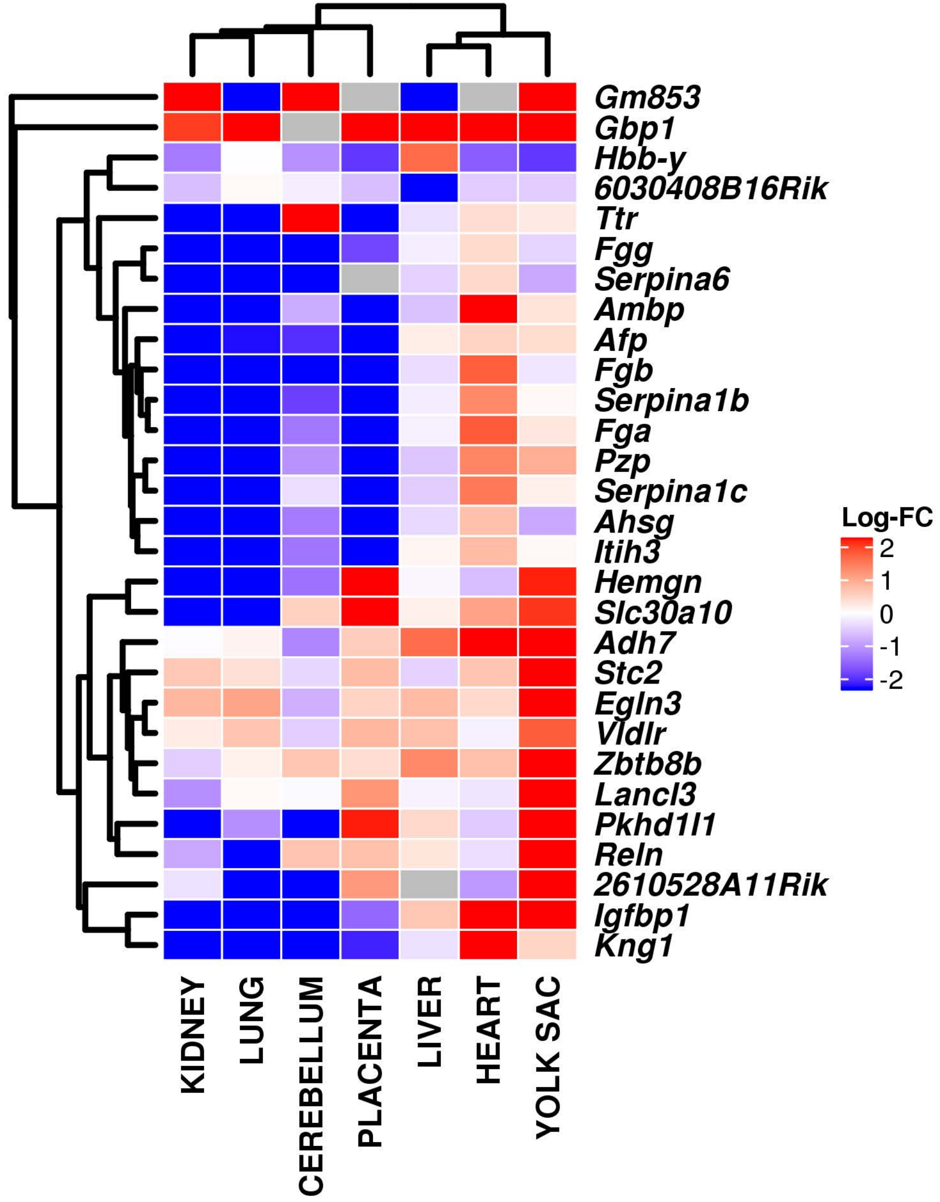

Gm853
Gbp1
Hbb-y
6030408B16Rik
Ttr
Fgg
Serpina6
Ambp
Afp
Fgb
Serpina1b
Fga
Pzp
Serpina1c
Ahsg
Itih3
Hemgn
Slc30a10
Adh7
Stc2
Egln3
Vldlr
Zbtb8b
Lancl3
Pkhd1l1
Reln
2610528A11Rik
Igfbp1
Kng1
KIDNEY
LUNG
CEREBELLUM
PLACENTA
LIVER
HEART
YOLK SAC
Log-FC
2
1
0
-1
-2
